# Optimizing HIV Spending Among Key Populations in Five Latin American and Caribbean Countries: A Mathematical Modelling Study

**DOI:** 10.1002/jia2.70171

**Published:** 2026-07-25

**Authors:** Debra ten Brink, Anna L. Bowring, Rowan Martin‐Hughes, Nisaa Wulan, Kelvin Burke, Cielo Yaneth Ríos Hincapie, Ricardo Luque Núñez, Brenda Cáceres‐Mejía, Rosa Victoria Sánchez, José Pablo Montoya Calvo, Sandra Margarita Nuñez Rubio, Maria Petro Brunal, Nick Scott

**Affiliations:** ^1^ Burnet Institute Melbourne Victoria Australia; ^2^ Ministerio de Salud y Protección Social Bogotá Colombia; ^3^ Facultad De Medicina Universidad Tecnológica del Perú Lima Perú; ^4^ Encargada de la División de Monitoreo y Evaluación de la Respuesta Nacional, Consejo Nacional para el VIH y el SIDA Santo Domingo Dominican Republic; ^5^ Ministerio de Salud San José Costa Rica; ^6^ Secretaria de Salud Tegucigalpa, MDC Honduras; ^7^ The Global Fund to Fight AIDS, Tuberculosis and Malaria Geneva Switzerland

**Keywords:** allocative efficiency, cost‐effectiveness, funding, HIV, key populations, mathematical modelling

## Abstract

**Introduction:**

From 2010 to 2024, the Latin America region experienced a 13% increase in new HIV acquisitions, while progress in reducing HIV acquisitions has slowed in the Caribbean. We conducted allocative efficiency studies for five countries in the Latin America and Caribbean region, aiming to estimate how HIV spending could be optimally allocated for maximal impact.

**Methods:**

Between 2021 and 2025, we collaborated with national partners in Colombia, Costa Rica, Dominican Republic, El Salvador and Honduras to develop national Optima HIV models incorporating country‐specific key populations. Prevention, testing and antiretroviral therapy (ART) interventions were parameterized using country‐specific unit costs, coverage estimates and effectiveness values. For each country, we projected HIV incidence, mortality and treatment resource needs from 2025 to 2030 for multiple scenarios: fixed intervention coverage (status quo), fixed spending allocations (counterfactual), and most recent spending optimized across interventions to minimize cumulative HIV acquisitions and HIV‐related deaths.

**Results:**

With the status quo continued, HIV incidence was projected to decline by 2030 in two countries, stabilize in two and increase in one country. Transgender women and men who have sex with men have the highest incidence rate. Due to ongoing increases in the number of diagnosed people living with HIV, by 2030, annual ART resources will need to increase by 1%–15% to maintain the existing treatment coverage.

Compared with the counterfactual (fixed spending) scenario, optimization of existing resources could avert 18%–33% of new HIV acquisitions and 5%–31% of HIV‐related deaths over 2025–2030 by prioritizing primarily ART. If modelled ART unit costs reduce by 25%, then 23%–69% of projected HIV acquisitions and 8%–61% of HIV‐related deaths could be averted by reinvesting savings to scale‐up interventions for country‐specific key populations instead of lower‐yield interventions such as untargeted HIV testing.

**Conclusions:**

This multi‐country modelling study demonstrates the potential to reduce new HIV acquisitions and HIV‐related deaths across Latin America and the Caribbean without increasing total spending. Recent reductions in treatment costs provide greater flexibility to invest in key population‐focused services which could amplify epidemic impact. The findings underscore the importance of aligning HIV resources with epidemic drivers and expanding targeted prevention for key populations.

## 1 Introduction

The Latin America region experienced a 13% increase in annual HIV acquisitions from 2010 to 2024, reaching 120,000 new HIV acquisitions in 2024 [1]. While the Caribbean has seen a 21% reduction in HIV acquisitions since 2010, progress has slowed, and incidence remains high and increasing in some settings [1]. Despite clear progress in expanding treatment access, gaps in service delivery persist, particularly among populations who are most affected by HIV. In 2022, in the Latin America and Caribbean regions, an estimated 57% and 33%, respectively, of people who newly acquired HIV were from key populations, including female sex workers, men who have sex with men, people who inject drugs, and transgender and gender diverse people [2]. These populations remain disproportionately affected by HIV due to persistent structural barriers, stigma and under‐resourced prevention strategies [3].

HIV prevalence among men who have sex with men is a median of 8.2% in Latin American countries and 11.3% in the Caribbean, and among transgender women nears 10% and 35%, respectively, far exceeding the general population estimates [3, 4]. Despite advances in HIV prevention options and Latin American countries contributing to trials and demonstration projects, coverage of pre‐exposure prophylaxis (PrEP) among key populations remains low and below regional targets, and prevention and treatment services may not be reaching groups most in need [3, 5]. Studies also report elevated HIV prevalence among Indigenous populations relative to the general population across multiple countries and communities [6, 7, 8].

Migration has further compounded regional HIV vulnerabilities. Since the economic and political collapse in Venezuela in 2014, more than 6.5 million Venezuelans have migrated across the region [9]. Displacement and migration increases HIV risks through vulnerability to sexual exploitation, limited healthcare access, financial barriers, insecure legal status and discrimination [10, 11]. This includes lack of access to HIV prevention options such as condoms or PrEP, decreased likelihood of a diagnosis and an increased risk of treatment interruption [12].

Country responses to HIV in Latin America and the Caribbean face several challenges related to financing. Domestic sources finance a majority of HIV spending in Latin America (an estimated 96% in 2024 [3]); however, they have not fully recovered from the impacts of COVID‐19 [13]. International sources, while a minority of overall HIV spending, predominantly fund HIV prevention tailored to key populations, making these programmes particularly vulnerable to recent shifts in global health funding [13]. Countries in the Caribbean rely more heavily on international resources for their HIV response [4], and funding cuts pose broad risks to progress. A 2025 analysis estimated that international funding cuts could increase the cumulative number of people from key populations acquiring HIV in Latin American and Caribbean countries by up to 150% by 2030 [14]. Further, reclassification of many Latin American countries as middle‐income reduces eligibility for international support mechanisms, including funding from the Global Fund and voluntary licensing agreements for more affordable access to HIV medicines such as lenacapavir [15].

Even if current spending levels could be maintained, many countries in Latin America are not on track to meet UNAIDS prevention and treatment targets [3]. It is crucial for countries to maximize the impact of all resources allocated to HIV. For five countries in the Latin America and Caribbean region, this study estimates how HIV spending can be optimized to minimize new HIV acquisitions and HIV‐related deaths, considering country‐specific diverse key population groups and epidemiological factors.

## 2 Methods

### 2.1 Settings and Stakeholder Involvement

National analyses were undertaken for Colombia, Costa Rica, Dominican Republic, El Salvador and Honduras. Country selection was opportunistic and based on independent Optima HIV analyses conducted between 2021 and 2025 in collaboration with Ministries of Health, National HIV/AIDS committees, the Global Fund, and, in some settings, civil society organizations.

For each analysis, country partners guided the inclusion of sub‐population groups and modelled interventions, contributed data inputs, and informed parameters such as the maximum expansion of interventions and constraints on resource allocation. Country‐validated models were finalized in 2021–2025 (Table 1) and updated in 2026 to Optima HIV version 2.11.4 or later and to include the latest publicly available treatment data up to 2024 [16]. Treatment spending had been excluded from the optimized budget in the original analyses of some countries to focus on prevention and testing priorities, and constraints on spending reallocation were individually defined. For improved comparability in this multi‐country analysis, all optimizations were updated to use consistent constraints (specified below), and to include antiretroviral therapy (ART) and prevention of mother‐to‐child transmission (PMTCT) in all budget optimizations.

**TABLE 1:** Overview of model specifications and baseline epidemic indicators.

	Colombia	Costa Rica	Dominican Republic	El Salvador	Honduras
**Model specifications**					
Year model finalized^a^	2025	2023	2023	2021	2021
Calibration source and version	Spectrum 2025	Spectrum 2025^b^	Spectrum 2019 via MISPAS [17]	SUMEVE, Spectrum 2020	Spectrum 2020
**Country overview (2024)**					
Country income status	Upper‐middle	High	Upper‐middle	Upper‐middle	Lower‐middle
Population size	52,300,000	5,200,000	10,600,000	6,600,000	10,800,000
**HIV epidemic overview (2024)** ^c^					
New HIV acquisitions	11,430	1090	1460	1020	770
HIV‐related deaths	3320	220	600	360	590
Number of people living with HIV	242,130	20,260	73,700	28,860	26,000
HIV prevalence among adults aged 15+ years (%)	0.6%	0.5%	0.9%	0.6%	0.3%
**Baseline progress towards 95‐95‐95 targets (2024)** ^c^					
Diagnosed people living with HIV (%)	79%	74%	90%	81%	68%
Diagnosed people on treatment (%)	82%	94%	84%	68%	78%
People on treatment with viral suppression (%)	93%	64%	89%	70%	80%
Baseline HIV spending^d^	$130,067,484	$13,086,135	$42,345,328	$15,513,102	$31,853,627

Abbreviations: M, million; MISPAS, Ministerio de Salud Pública y Asistencia Social (Dominican Republic); SUMEVE, El Sistema Único de Monitoreo y Evaluación y Vigilancia Epidemiológica del VIH (El Salvador).

^a^ Year when country‐validated model was finalized. Excludes additional updates made as part of this multi‐country analysis.

^b^ Model originally calibrated to Spectrum 2022. Due to Optima HIV model updates and significant changes to Spectrum estimates, the calibration was updated to better reflect Spectrum 2025 estimates while retaining existing intervention definitions.

^c^ Optima‐modelled values. These may differ to current Spectrum estimates due to differences in model structure and assumptions and the year the model was finalized.

^d^ Estimated based on direct and targeted HIV spending and reported in 2024 US$. Excludes private spending on condoms in El Salvador and Honduras, which was also excluded from optimizable spending in original country analyses.

*Source*: Optima HIV modelled values for population size (aligned to World Population Prospects at time of analysis or country preferred source), HIV epidemic and cascade indicators. Income status based on the World Bank [18].

### 2.2 Optima HIV Model

These analyses applied Optima HIV models. In brief, Optima HIV is a dynamic compartmental model of HIV transmission, with compartments stratified by setting‐specific population disaggregation (by age, sex and acquisition risks), disease progression and care cascade progression (Supplementary Appendix A, Tables A1 and A2) [19]. The epidemic model is linked to an economic optimization framework which, using country‐specific cost−coverage and coverage−impact relationships, identifies how defined budgets can be optimally allocated to maximize epidemic gains.

### 2.3 Population Groups

Each country model included epidemiologically relevant sub‐populations defined by country teams (Table 2 and Tables B1–B5). Key and priority populations modelled included female sex workers, clients of female sex workers, men who have sex with men, transgender women, people who inject drugs, people in prison or other closed settings, migrants and Indigenous populations. Non‐key populations aged 0–99 years were modelled by sex and country‐defined age groups (e.g. 0–14, 15–49 and 50+ years).

**TABLE 2:** Populations and HIV interventions included in the analysis for each of the five countries.

	Colombia	Costa Rica	Dominican Republic	El Salvador	Honduras
**Population groups**					
Female sex workers (FSWs)	X	X	X	X	X
Clients of FSWs	X	X	X	X	X
Men who have sex with men (MSM)	X	X	X	X	X
Transgender women (TGW)	X	X	X	X	X
Migrants	X		X		
Indigenous populations					X
People who inject drugs (PWID)	X				
People who use drugs			X^a^		
People in prisons and other closed settings	X	X	X	X	X
People experiencing homelessness	X				
Non‐key populations (aged 0–99)	X	X	X	X	X
**HIV interventions**					
Antiretroviral therapy (ART)	X	X	X	X	X
Prevention of mother‐to‐child transmission (PMTCT)	X	X	X	X	X
Treatment support programmes			X		
HIV testing for the general population	X	X^b^	X	X	X
Condom distribution/promotion among the general population^c^	X	X	X	X	X
HIV self‐testing	X				
HIV services for FSWs^d^	X	X	X	X	X
HIV services for MSM^d^	X	X	X	X	X
HIV services for TGW^d^	X	X	X	X	X
HIV services for PWID^d^	X				
HIV services for migrants^d^	X		X		
HIV services for people in prisons^d^	X	X	X	X	X
PrEP for MSM	X^e^	X	X		
PrEP for TGW	X^e^				
PrEP for FSWs	X^e^		X		
PrEP for migrants			X		
Post‐exposure prophylaxis (PEP)				X	

Abbreviations: ART, antiretroviral therapy; FSWs, female sex workers; MSM, men who have sex with men; PEP, post‐exposure prophylaxis; PMTCT, prevention of mother‐to‐child (vertical) transmission; PrEP, pre‐exposure prophylaxis; PWID, people who inject drugs; TGW, transgender women; X, included in country model.

^a^ Includes people who use drugs through injecting and non‐injecting routes.

^b^ General HIV testing programme modelled to reach all populations in Costa Rica, and key‐population services include prevention only.

^c^ Condom distribution and promotion reaching non‐key populations (i.e. not focused on modelled key population groups).

^d^ Includes HIV prevention (excluding PrEP) and testing services tailored to key population groups except for Costa Rica (HIV prevention only).

^e^ Separate modalities modelled for oral and long‐acting PrEP.

### 2.4 HIV Programmes

Programmes covered a spectrum of prevention, testing and treatment interventions: prevention services tailored to key populations, HIV testing modalities, condom distribution, PrEP for key populations, PMTCT, ART and treatment support (Table 2). Interventions were parameterized using country‐specific unit costs, coverage estimates and effectiveness values at the time the studies were conducted from National AIDS Spending Assessments, Global Fund budgets and domestic financing reports (Tables B6–B10). Cost−coverage relationships for programmes are non‐linear above individually defined coverage thresholds, to account for higher marginal costs at high coverage as the remaining accessible population decreases.

### 2.5 Data Inputs

Each country model was parameterized with: epidemiological data (population size estimates, HIV prevalence, incidence, CD4 distributions and mortality) obtained from national surveillance reports, UNAIDS Spectrum files and peer‐reviewed literature; behavioural data (condom use, number of sexual partners, injecting frequency, needle and syringe sharing, testing rates) from Integrated Biological and Behavioral Surveys (IBBS), Demographic and Health Surveys, and country‐specific key population studies (Table A3 and Tables B1–B5). Country models were calibrated to the latest Spectrum or country‐preferred estimates (Table 1 and Figure C‐1).

### 2.6 Costs

Costs associated with HIV prevention, testing and treatment programmes were considered from a health system perspective (i.e. including government and international donor spending and spending from social health insurance schemes). Only costs that could be directly attributed to HIV programmes were included (i.e. excluding non‐targeted spending such as infrastructure, management, research and services related to coinfections and opportunistic infections).

Most recent spending on HIV programmes reflects the latest available estimates obtained for each country at the time the studies were conducted (between 2021 and 2025), adjusted to account for ART and PMTCT coverage updated to 2024, inclusive. Spending was adjusted to 2024 US$ based on the consumer price index and presented without discounting [20].

### 2.7 Scenarios

For each country, this analysis considers outcomes from four scenarios over 2025–2030 inclusive:

1. Status quo: fixed coverage of all HIV prevention and testing services maintained until 2030, and treatment coverage maintained at fixed proportion of people with diagnosed HIV, based on proportional coverage in year of most recent data;
2. Counterfactual (fixed spending): fixed spending allocation until 2030 based on most recent spending on HIV prevention, testing and treatment services;
3. Optimized spending: 100% of the most recent spending reallocated across HIV interventions in 2025 to minimize cumulative new HIV acquisitions and HIV‐related deaths by 2030;
4. Optimized spending with reduced ART cost: scenario 3 but with a theoretical 25% reduction in ART unit costs for the projected period 2025–2030.

Each optimization assumes spending is reallocated in 2025 and maintained until 2030.

The difference between the status quo and counterfactual (fixed spending) scenarios is the assumption about maintaining either fixed proportional coverage or fixed spending. The status quo can be interpreted as projecting continued current trends. The counterfactual (fixed spending) scenario serves as a technical comparator for the optimized spending scenario to isolate the impact of reallocation under a fixed resource constraint. Under this assumption, the absolute number of people on treatment will stay the same each year, but as more diagnoses are made through continued testing, the proportion of people diagnosed who are on treatment reduces over time. Without this scenario, it would not be possible to attribute improvements under optimized spending to reallocation decisions rather than to changes in total spending.

In the optimized spending scenarios, constraints were applied for all countries such that no one was removed from ART or PMTCT unless by natural attrition. A minimum of 50% of funding was required to be retained for existing interventions to allow flexibility in reallocation while preventing the loss of the majority (>50%) of funding, given equity considerations, logistical constraints and potential non‐HIV‐related benefits of each programme. The optimization objective weighted minimizing HIV acquisitions and HIV‐related deaths as 1−5. Standard Optima analyses recommend using this weighting to produce a balanced optimization in line with policy objectives. Alternative objective weightings are presented in Supplementary Appendix C (Tables C2–C4).

Costs for first‐line HIV treatment have notably decreased over the years in low‐ and middle‐income countries due to access to generic medicines through voluntary licensing agreements and waivers and use of pooled procurement and supply management systems [3, 21, 22]. Most recent treatment cost estimates in these five analyses were derived from 2019 to 2022 data, which was prior to the latest reductions. The Global Fund Agreements announced in 2023 anticipated a 25% reduction in costs of first‐line treatment with tenofovir disoproxil fumarate, lamivudine and dolutegravir (TLD) [23], while Pan American Health Organization (PAHO) Strategic Fund reference prices for dolutegravir and TLD reduced by 32%–52% from 2022 to 2025 [24, 25]. Colombia issued a compulsory license for dolutegravir in 2023, enabling purchase of cheaper generic antiretrovirals since 2024 [26]. Ongoing reductions in costs may also be plausible through earlier diagnosis of HIV with treatment initiation [27, 28]. Since ART is the largest contributor to total budgets, scenario 4 was constructed as a univariate sensitivity analysis to examine whether a hypothetical 25% reduction in estimated ART unit costs, representing both plausible reductions since modelled HIV treatment costs were defined and achievable reductions through a combination of factors, could ensure higher coverage for other interventions, different intervention prioritizations, and differences in HIV acquisitions and HIV‐related deaths averted.

### 2.8 Outcomes

For comparability in the multi‐country analysis, HIV interventions and associated spending were consolidated into 13 HIV intervention categories. Absolute and relative changes in spending were calculated between most recent and optimized spending allocations by country. To quantify uncertainty in the model projections, we generated 100 plausible baseline projections by sampling the key calibration parameters (Supplementary Appendix C); each scenario was applied onto these plausible parameter sets.

The cumulative number of new HIV acquisitions and HIV‐related deaths over 2025−2030, inclusive, were compared between the optimized spending and counterfactual (fixed spending) scenarios. Additional outcomes assessed were the additional resources required for HIV treatment to maintain treatment coverage over 2025−2030 in the status quo scenario and the projected treatment cascade in 2030 for each scenario reported in relation to 95‐95‐95 targets.

### 2.9 Ethical Considerations and Data Permissions

All analyses relied exclusively on aggregated secondary data from publicly available or country‐shared reports and did not require institutional review board approval. Each participating Ministry of Health reviewed and approved the use of national data for modelling and publication.

## 3 Results

### 3.1 Status Quo Scenario

**FIGURE 1:**
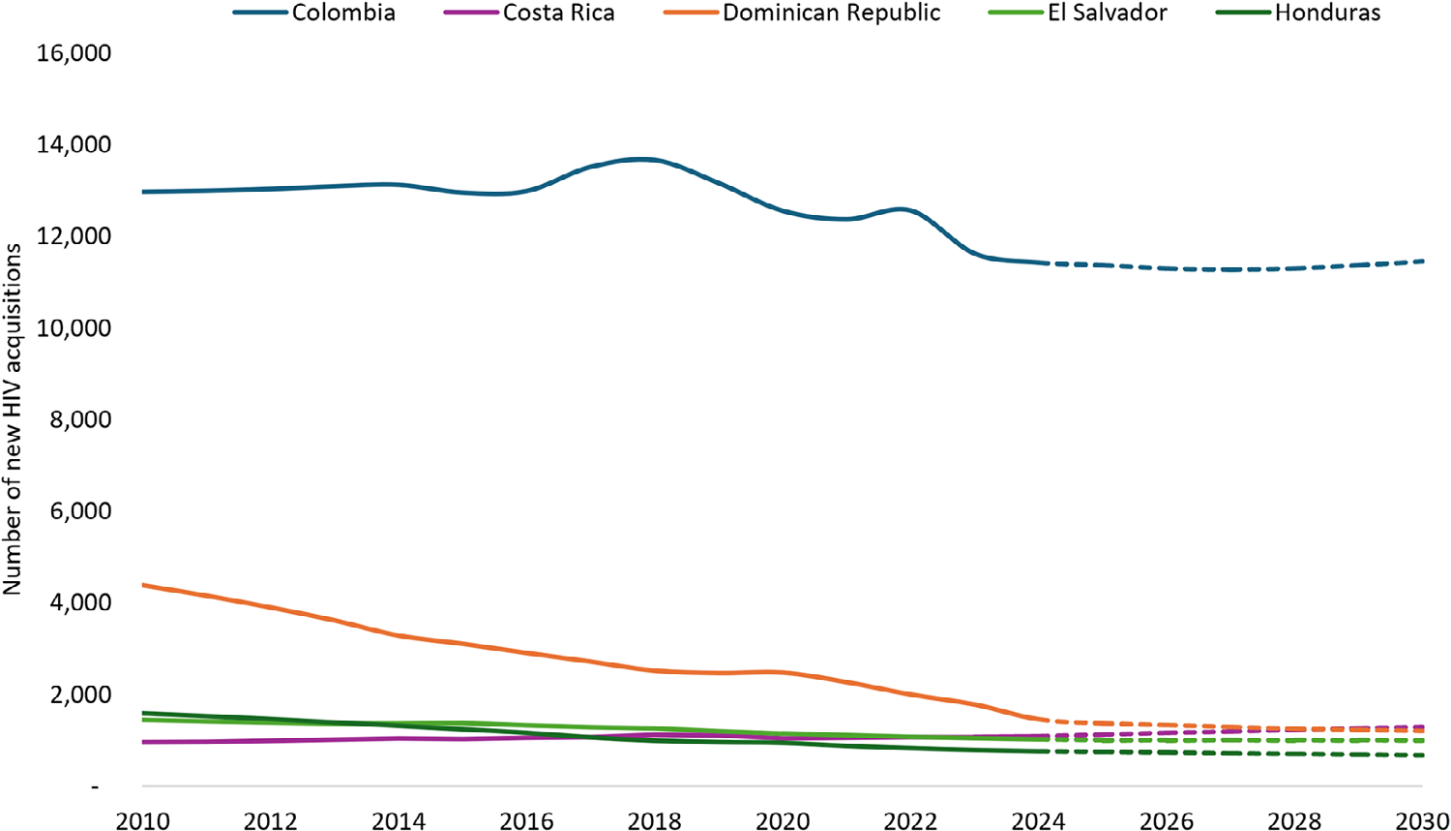
Model‐projected total new HIV acquisitions under the status quo scenario, 2010−2030 for Colombia, Costa Rica, El Salvador, Dominican Republic and Honduras. Solid line represents historical trends calibrated to HIV prevalence trends and overall new HIV acquisitions from Spectrum estimates given treatment data up to 2024. Dashed line designates beginning of model projections (2025).

**FIGURE 2:**
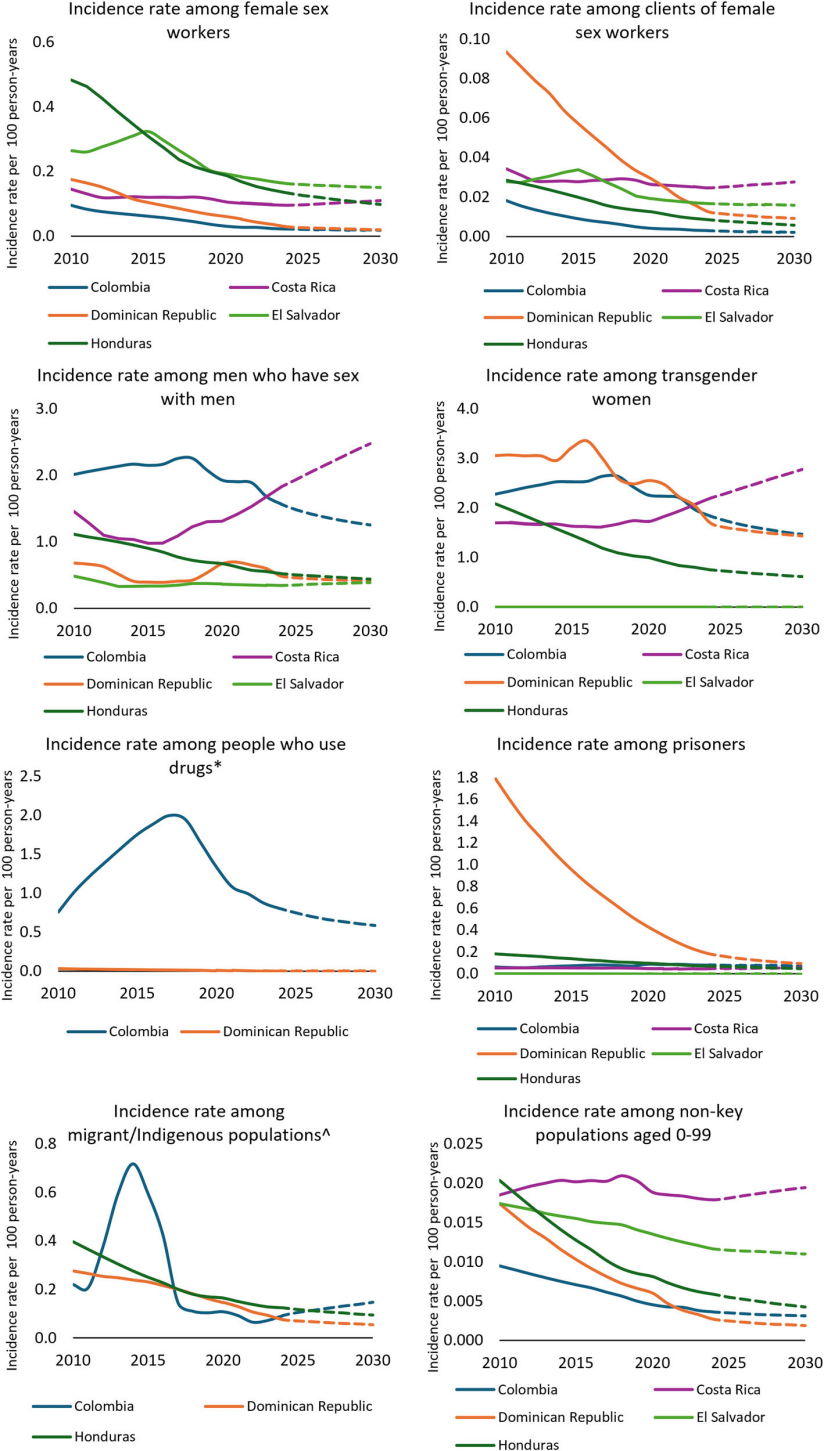
Model‐projected incidence rates per 100 person‐years from 2010 to 2030 by key population group in Colombia, Costa Rica, Dominican Republic, El Salvador and Honduras under the status quo scenario. * Based on people who inject drugs in Colombia and people who use drugs through injecting and non‐injecting routes in the Dominican Republic. ^ Migrant and Indigenous populations consider Venezuelan migrants in Colombia, Haitian migrants in the Dominican Republic and the Garifuna population in Honduras. Solid line represents historical trends calibrated to HIV prevalence trends and overall new HIV acquisitions from Spectrum estimates given treatment data up to 2024. Dashed line designates beginning of model projections (2025).

Total annual new HIV acquisitions are projected to decline or stabilize from 2025 to 2030 in four countries, assuming the treatment coverage can continue at the existing proportion of people with diagnosed HIV, but could increase in Costa Rica (15%; Figure 1) as well as some population groups (Figure 2). By population, incidence rate was highest among transgender women in all countries except for El Salvador (Figure 2). Model projections indicate that in 2030 transgender women and men who have sex with men are likely to remain the populations most affected by HIV.

### 3.2 Resources to Maintain Treatment Coverage

The status quo scenario projects that by 2030 the number of diagnosed people living with HIV will have increased by 1%–28% in participating countries compared to 2025. Therefore, maintaining treatment coverage will require annual spending on ART to proportionally increase if there is no change to the existing unit cost (Table 3). Comparatively, in the counterfactual (fixed spending) scenario where there is no additional spending on treatment (or no further reductions in ART unit costs), then ART coverage could reduce as the denominator increases.

**TABLE 3:** Resources required for ART with fixed spending (counterfactual scenario) compared to fixed proportional coverage (status quo scenario).

	Percentage increase in number of diagnosed people living with HIV, 2025−2030	ART coverage by 2030 (% of diagnosed people living with HIV)		Total ART resources required, 2025–2030		Additional resources required to maintain ART coverage, 2025–2030	
		Fixed spending^a^	Fixed ART coverage^b^	Fixed spending^a^	Fixed ART coverage^b^	*n*	%
Colombia	17%	67%	82%	$650.84 M	$742.17 M	$91.3 M	14%
Costa Rica	28%	74%	94%	$46.29 M	$53.23 M	$6.9 M	15%
Dominican Republic	1%	86%	84%	$152.36 M	$154.02 M	$1.7 M	1%
El Salvador	7%	63%	68%	$29.33 M	$30.87 M	$1.5 M	5%
Honduras	9%	72%	78%	$122.83 M	$132.06 M	$9.2 M	8%

*Note*: All spending shown in 2024 US$ (millions, M).

Abbreviation: ART, antiretroviral therapy.

^a^ Based on most recent spending estimates derived from country studies adjusted to account for number on treatment updated to 2024.

^b^ Based on Optima modelled ART coverage among diagnosed people living with HIV in 2024.

### 3.3 Counterfactual (Fixed Spending) Scenario

Across five countries, the counterfactual (fixed spending) scenario included US$13.1–$130.1 million in annual HIV spending over 2025–2030. Treatment accounted for between 32% (El Salvador) and 84% (Colombia) of total HIV spending. Between 4% (Colombia) and 40% (El Salvador) of total HIV spending was spent on key and priority population‐focused testing and prevention services, including PrEP.

In the counterfactual (fixed spending) scenario, a range of 4921–96,457 new HIV acquisitions and 2183–27,854 HIV‐related deaths were projected over 2025–2030 across five countries. Incidence rates were projected to increase over 2025–2030 more widely among key populations, including among men who have sex with men (4/5 countries), transgender women (3/5), female sex workers (2/5), people who use drugs (1/2), people in prison (2/5), and migrant and Indigenous populations (1/3). Conversely, HIV incidence among non‐key populations (all ages) was only projected to increase in Costa Rica (Figure C‐2).

In the counterfactual (fixed spending) scenario, by 2030 countries were projected to reach 74%–95% of people living with HIV diagnosed, 63%–86% of diagnosed people on treatment and 64%–93% of people on treatment with viral suppression relative to 95‐95‐95 targets.

### 3.4 Optimized Spending Scenario

**FIGURE 3:**
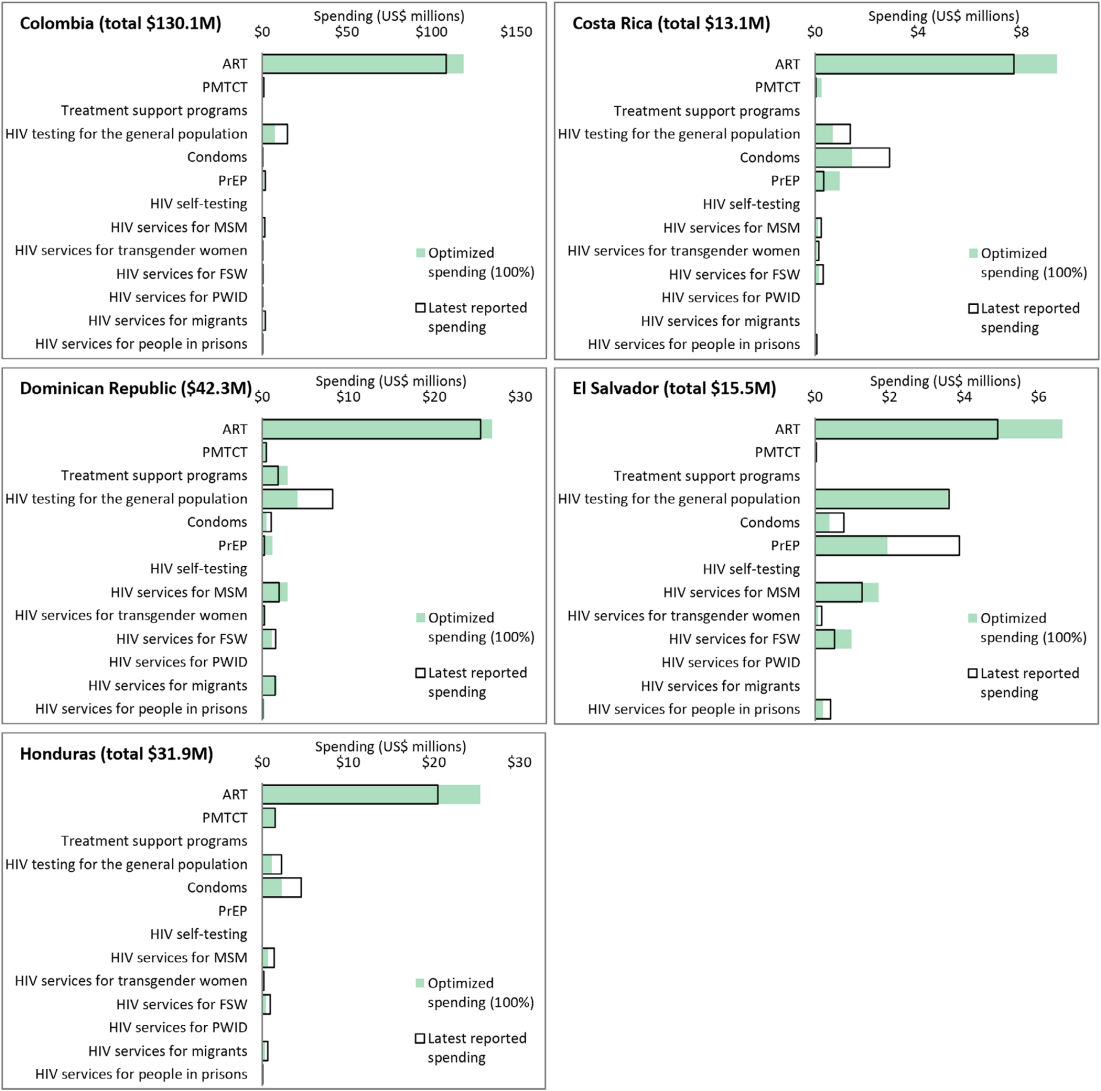
Latest reported spending (black outline) and optimized spending allocation (green bar) to minimize new HIV acquisitions and HIV‐related deaths by country. ART, antiretroviral therapy; Condoms, condoms distribution and promotion among general population; FSWs, female sex workers; MSM, men who have sex with men; PMTCT, prevention of mother‐to‐child (vertical) transmission; PrEP, pre‐exposure prophylaxis (includes post‐exposure prophylaxis in El Salvador); PWID, people who inject drugs. HIV services for key populations include condom‐based prevention, social and behaviour change communication, and HIV testing. Spending reported in 2024 US$ millions.

In all five countries, the optimization prioritized increased spending on ART to maintain or increase current treatment coverage (Figure 3, Table 4 and Table C‐1). Spending for key populations was also prioritized for scale‐up on a country‐specific basis, including men who have sex with men (2/5 countries), transgender women (1/5), female sex workers (1/5), migrants or Indigenous populations (1/3), PrEP (2/4) and people in prisons (1/5). These increases in spending were achieved by reallocating resources away from broad, lower‐yield interventions such as condom distribution and HIV testing for the general population.

**TABLE 4:** Change in spending allocation by intervention and country with 100% spending optimized.

		Colombia	Costa Rica	Dominican Republic	El Salvador	Honduras
ART		+$10,397,000 (10%)	+$1,658,000 (21%)	+$1,315,000 (5%)	+$1,735,000 (36%)	+$4,933,000 (24%)
PMTCT		No change	+$221,000 (596%)	No change	No change	No change
Treatment support programmes		NA	NA	+$1,111,000 (60%)	NA	NA
HIV testing for the general population^a^		−$7,477,000 (−50%)	−$680,000 (−50%)	−$4,088,000 (−50%)	−$23,000 (−1%)	−$1,128,000 (−50%)
Condom distribution/promotion among the general population		−$85,000 (−50%)	−$1,440,000 (−50%)	−$519,000 (−50%)	−$387,000 (−50%)	−$2,264,000 (−50%)
PrEP^b^		−$912,000 (−50%)	+$625,000 (192%)	+$972,000 (426%)	−$1935,000 (−50%)	NA
HIV self‐testing		−$21 (−50%)	NA	NA	NA	NA
HIV prevention and testing services for MSM^c^		−$767,000 (−50%)	−$123,000 (−50%)	+$994,000 (51%)	+$445,000 (36%)	−$683,000 (−50%)
HIV prevention and testing services for transgender women^c^		−$50,000 (−50%)	−$74,000 (−50%)	+$157,000 (70%)	−$86,000 (−50%)	−$81,000 (−50%)
HIV prevention and testing services for FSWs^c^		−$190,000 (−50%)	−$154,000 (−50%)	−$432,000 (−28%)	+$459,000 (89%)	−$455,000 (−50%)
HIV prevention and testing services for PWID		−$68,000 (−50%)	NA	NA	NA	NA
HIV prevention and testing services for migrants/Indigenous populations		−$843,000 (−50%)	NA	+$230,000 (16%)	NA	−$310,000 (−50%)
HIV prevention and testing services for people in prisons		−$6000 (−50%)	−$33,000 (−50%)	+$259,000 (20273%)	−$209,000 (−50%)	−$14,000 (−50%)
	KEY					
		Increased spending prioritized				
		Spending maintained (±2%)				
		Decreased spending				
		Not modelled				

*Note*: All spending rounded to the nearest 1000 and reported in 2024 US$.

Abbreviations: ART, antiretroviral therapy; FSWs, female sex workers; MSM, men who have sex with men; PMTCT, prevention of mother‐to‐child (vertical) transmission; PrEP, pre‐exposure prophylaxis (includes post‐exposure prophylaxis in El Salvador); PWID, people who inject drugs.

^a^ General HIV testing modelled to reach all populations in Costa Rica.

^b^ Based on post‐exposure prophylaxis in El Salvador.

^c^ Key‐population focused programmes exclude testing in Costa Rica.

Compared to their counterfactual (fixed spending) scenarios, with HIV spending optimized 18%–33% of HIV acquisitions and 5%–31% of HIV‐related deaths could be averted over 2025–2030 across countries (Table 5 and Figure C‐3). Optimized spending increases progress on ART coverage.

**TABLE 5:** Impact of 100% spending optimized on cumulative new HIV acquisitions and HIV‐related deaths from 2025 to 2030 and treatment cascade indicators in 2030 by country.

		Colombia	Costa Rica	Dominican Republic	El Salvador	Honduras
**Cumulative new HIV acquisitions, 2025**–**2030**						
Counterfactual (fixed spending)	Number [95% CI]	96,457 [52,790, 154,892]	9362 [6726, 14,843]	6999 [5331, 9880]	6326 [5245, 7665]	4921 [3896, 6061]
100% optimized	Number [95% CI]	79,046 [38,865, 139,262]	6943 [5686, 10,651]	4691 [4018, 6544]	4965 [4336, 5900]	3813 [3127, 4534]
	Difference, *n* (%) [95% CI] ^a^	−17,411 (−18%) [−17,720, −13,436]	−2419 (−26%) [−4156, −1002]	−2308 (−33%) [−3410, −1327]	−1361 (−22%) [−1799, −872]	−1108 (−23%) [−1508, −777]
**Cumulative HIV‐related deaths, 2025**–**2030**						
Counterfactual (fixed spending)	Number [95% CI]	27,854 [15,661, 43,972]	2215 [1544, 3645]	2469 [2175, 2899]	2183 [1875, 2606]	3838 [3045, 4639]
100% optimized	Number [95% CI]	21,506 [10,943, 37,197]	1539 [1310, 2442]	2335 [2116, 2714]	1974 [1748, 2282]	2878 [2502, 3195]
	Difference, *n* (%) [95% CI] ^a^	−6348 (−23%) [−6895, −4761]	−676 (−31%) [−1203, −237]	−134 (−5%) [−186, −60]	−209 (−10%) [−325, −126]	−960 (−25%) [−1461, −542]
**Treatment cascade indicators (95‐95‐95 targets), 2030**						
Counterfactual (fixed spending)	Diagnosed people living with HIV	80%	74%	95%	84%	74%
100% optimized		80%	77%	96%	86%	76%
Counterfactual (fixed spending)	Diagnosed people living with HIV on treatment	67%	74%	86%	63%	72%
100% optimized		76%	90%	91%	83%	87%
Counterfactual (fixed spending)	People on treatment with viral suppression^b^	93%	64%	89%	70%	80%
100% optimized		93%	64%	89%	70%	80%

Abbreviations: ART, antiretroviral therapy; CI, confidence interval; *n*, number.

^a^ Difference from counterfactual (fixed spending) scenario, that is number of new HIV acquisitions/deaths averted.

^b^ No programmes were modelled to have a direct impact on viral suppression, and viral suppression was assumed to remain fixed at the most recent estimate. In the Dominican Republic, the treatment support programme was modelled to reduce loss‐to‐follow‐up but does not directly impact viral suppression among those on treatment.

### 3.5 Optimized Spending With Reduced ART Costs Scenario

If the modelled unit cost of ART reduced by 25% in participating countries, ART was maintained in 3/5 countries and reduced elsewhere, allowing greater reallocation of resources to HIV prevention and testing services tailored to key populations: men who have sex with men (5/5 countries) and transgender women (4/5), female sex workers (2/5), people who inject drugs (1/1), migrants or Indigenous populations (1/3), HIV self‐testing (1/1) and PrEP (2/4) (Table 6 and Table C‐1).

**TABLE 6:** Change in spending allocation by intervention and country with 100% spending optimized with reduced ART costs.^a^

		Colombia	Costa Rica	Dominican Republic	El Salvador	Honduras
ART^a^		−$38,000 (0%)	−$637,000 (−8%)	−$5,051,000 (−20%)	+$80,000 (2%)	+$342,000 (2%)
PMTCT		No change	+$212,000 (573%)	No change	No change	+$1,583,000 (104%)
Treatment support programmes		NA	NA	+$2,602,000 (140%)	NA	NA
HIV testing for the general population^b^		−$7,477,000 (−50%)	+$823,000 (61%)	−$4,088,000 (−50%)	+$493,000 (14%)	−$1,128,000 (−50%)
Condom distribution/promotion among the general population		−$85,000 (−50%)	−$1,440,000 (−50%)	−$519,000 (−50%)	−$387,000 (−11%)	−$2,264,000 (−18%)
PrEP^c^		−$912,000 (−50%)	+$758,000 (233%)	+$1,457,000 (639%)	−$1,935,000 (−50%)	NA
HIV self‐testing		+$822,000 (1,966,153%)	NA	NA	NA	NA
HIV prevention and testing services for MSM^d^		+$7,932,000 (517%)	+$328,000 (133%)	+$2,558,000 (133%)	+$680,000 (54%)	+$1,869,000 (137%)
HIV prevention and testing services for transgender women^d^		+$113,000 (115%)	+$109,000 (74%)	+$368,000 (163%)	−$86,000 (−50%)	+$332,000 (205%)
HIV prevention and testing services for FSWs^d^		−$190,000 (−50%)	−$120,000 (−39%)	+$191,000 (12%)	+$1,363,000 (263%)	−$455,000 (−50%)
HIV prevention and testing services for PWID		+$294,000 (216%)	NA	NA	NA	NA
HIV prevention and testing services for migrants/Indigenous populations		−$843,000 (−50%)	NA	+$1,992,000 (136%)	NA	−$310,000 (−50%)
HIV prevention and testing services for people in prisons		+$382,000 (3049%)	−$33,000 (−50%)	+$490,000 (38302%)	−$209,000 (−50%)	+$29,000 (107%)
	KEY					
		Increased spending prioritized				
		Spending maintained (±2%)				
		Decreased spending				
		Not modelled				

*Note*: All spending rounded to the nearest 1000 and reported in 2024 US$.

Abbreviations: ART, antiretroviral therapy; FSWs, female sex workers; MSM, men who have sex with men; PMTCT, prevention of mother‐to‐child (vertical) transmission; PrEP, pre‐exposure prophylaxis (includes post‐exposure prophylaxis in El Salvador); PWID, people who inject drugs.

^a^ Assuming modelled unit cost of ART reduced by 25%.

^b^ General HIV testing modelled to reach all populations in Costa Rica.

^c^ Based on post‐exposure prophylaxis in El Salvador.

^d^ Key‐population focused programmes exclude testing in Costa Rica.

With reduced ART costs, optimizing most recent resources would lead to a greater epidemic impact, and between countries 23%–69% of new HIV acquisitions and 8%–61% of HIV‐related deaths could be averted over 2025–2030 compared to their counterfactual (fixed spending) scenarios (Table 7 and Figure C‐4).

**TABLE 7:** Impact of 100% spending optimized with reduced ART costs on cumulative new HIV acquisitions and HIV‐related deaths from 2025 to 2030 and treatment cascade indicators in 2030 by country.

		Colombia	Costa Rica	Dominican Republic	El Salvador	Honduras
**Cumulative new HIV acquisitions, 2025**–**2030**						
Counterfactual (fixed spending)	Number [95% CI]	96,457 [52,790, 154,892]	9362 [6726, 14,843]	6999 [5331, 9880]	6326 [5245, 7665]	4921 [3896, 6061]
100% optimized (reduced ART costs^a^)	Number [95% CI]	30,088 [24,302, 69,626]	6737 [5512, 10,354]	4265 [3670, 5999]	4865 [4249, 5789]	3069 [2595, 3641]
	Difference, *n* (%) [95% CI] ^b^	−66,369 (−69%) [−86,132, −28,362]	−2625 (−28%) [−4453, −1175]	−2734 (−39%) [−3949, −1676]	−1461 (−23%) [−1913, −957]	−1852 (−38%) [−2407, −1313]
**Cumulative HIV‐related deaths, 2025**–**2030**						
Counterfactual (fixed spending)	Number [95% CI]	27,854 [15,661, 43,972]	2215 [1544, 3645]	2469 [2175, 2899]	2183 [1875, 2606]	3838 [3045, 4639]
100% optimized (reduced ART costs^a^)	Number [95% CI]	10,876 [9187, 23,521]	1523 [1297, 2415]	2267 [2057, 2633]	1967 [1742, 2274]	2755 [2434, 3032]
	Difference, *n* (%) [95% CI] ^b^	−16,978 (−61%) [−21,041, −6600]	−692 (−31%) [−1231, −251]	−202 (−8%) [−267, −118]	−216 (−10%) [−333, −133]	−1083 (−28%) [−1630, −610]
**Treatment cascade indicators (95‐95‐95 targets), 2030**						
Counterfactual (fixed spending)	Diagnosed people living with HIV	80%	74%	95%	84%	74%
100% optimized (reduced ART costs^a^)		91%	79%	97%	86%	79%
Counterfactual (fixed spending)	Diagnosed people living with HIV on treatment	67%	74%	86%	63%	72%
100% optimized (reduced ART costs^a^)		92%	90%	91%	83%	94%
Counterfactual (fixed spending)	People on treatment with viral suppression^c^	93%	64%	89%	70%	80%
100% optimized (reduced ART costs^a^)		93%	64%	89%	70%	80%

Abbreviations: ART, antiretroviral therapy; CI, confidence interval; *n*, number.

^a^ Assumes 25% reduction in modelled unit cost of ART for all countries.

^b^ Difference from counterfactual (fixed spending) scenario, that is number of new HIV acquisitions/deaths averted.

^c^ No programmes were modelled to have a direct impact on viral suppression, and viral suppression was assumed to remain fixed at the most recent estimate. In the Dominican Republic, the treatment support programme was modelled to reduce loss‐to‐follow‐up but does not directly impact viral suppression among those on treatment.

## 4 Discussion

The HIV epidemic in the Latin America and Caribbean region remains an evolving public health challenge, particularly among key populations. This analysis found that countries will need to increase resources designated to HIV treatment to at least maintain current treatment coverage, as the number of people diagnosed with HIV continues to grow, but recent reductions in treatment costs likely afford greater flexibility to invest in prevention and testing. For the five participating countries in the region, focusing existing resources on the most‐affected population groups could further reduce the number of people acquiring HIV and HIV‐related deaths at a country level. The groups to prioritize differed by country, highlighting the importance of locally tailored responses.

Despite substantial improvements in treatment coverage among people living with HIV, scaling up ART was still the first priority for optimized spending across the five participating countries to maintain the historical gains in improving treatment coverage as the number of diagnosed people living with HIV grows. However, it is likely that treatment costs have reduced since they were defined in the country modelling analyses [23, 24, 25], and there may be several opportunities to further reduce ART costs. Rollout of differentiated service delivery for HIV treatment has been slow in Latin America compared to other regions [29], and strategies such as multi‐month dispensing may reduce health system costs associated with ART. Effective expansion of prevention and testing through optimized allocations may lead to earlier diagnoses and subsequently reduce care costs associated with later‐stage diagnoses [27, 28]. The hypothetical 25% reduction in ART unit costs, which may be more aligned with current treatment costs, could allow countries to increase ART coverage while continuing to provide and expand HIV services to key populations, thus amplifying projected epidemic impact.

With optimized spending assuming reduced ART costs, four countries are projected to exceed 90% treatment coverage among diagnosed people living with HIV by the end of 2030, compared to only one country at the 2024 baseline. Only two countries may also exceed or be in reach of targets for 95% diagnosis with optimized spending. Additional progress may be possible with either more resources available, new or innovative modalities for delivering services and technical efficiency gains in existing services. Modelled programmes did not capture specific testing and outreach modalities, such as physical and virtual outreach, social network‐based testing and services tailored to specific sub‐populations, and self‐testing was only modelled in one country. These are recommended in global guidelines [30], but further data are needed to explore the complementary value of such modalities locally and inform more granular decision‐making.

HIV services focused on the needs of key and priority populations accounted for less than 13% of targeted HIV spending in four countries. Transgender women and men who have sex with men had the highest prevalence in all countries and increasing incidence trends under the counterfactual (fixed spending) scenario. The sustained vulnerability of these populations highlights the ongoing structural barriers such as stigma, discrimination and criminalization that continue to constrain access to prevention and treatment services [31, 32]. Services reaching men who have sex with men and transgender women were prioritized for expansion in nearly all settings under the optimization scenario with reduced ART costs, but achieving this may require structural barriers to be addressed.

Other prioritized interventions and populations were more divergent across countries, reflecting differences in epidemic profiles, potential for expansion and programme effectiveness. HIV services for female sex workers were prioritized in El Salvador, where incidence is elevated and existing coverage was low. Prevention and testing services were prioritized for prisoners in the Dominican Republic to protect the significant gains made over the last decade. The consistent deprioritization of general‐population testing and condom distribution indicates limited marginal benefit from broad, untargeted prevention approaches, echoing global analyses that demonstrate diminishing returns from non‐targeted interventions in concentrated epidemics [33, 34]. However, these services may still play an important role for antenatal screening, surveillance and reaching people who either do not self‐identify or who avoid key population‐focused services due to stigma.

Migrant and Indigenous populations were explicitly modelled as priority populations in three of five countries, although interventions tailored for these groups were limited. Despite migrant and Indigenous populations being increasingly recognized as at heightened risk, national HIV strategies often lack specific, actionable plans for these populations. Strengthened data systems and routine outcome disaggregation could inform more targeted and cost‐effective interventions for these groups. As seen in Colombia with the establishment of Temporary Protection Status in 2021 [35], legal conditions and policy can facilitate access to mainstream health services among migrants, which may reduce optimization priorities for migrant‐specific services. However, interpretation of these findings should consider potential changes to immigration policy, delays to achieving protection status and other barriers to accessing health services experienced by migrants.

This analysis reports on varied epidemic contexts from five countries, but findings are not intended to be representative of the broader Latin America and Caribbean region. In particular, several populous countries with relatively large HIV epidemics are not included, such as Brazil and Haiti [16]. The following limitations could have led to the relative benefits of optimization being overstated, and results should be interpreted accordingly. Optimization is modelled as a single reallocation of spending in 2025 maintained through 2030, which may not reflect real‐world decision‐making, changes in underlying risk or practical constraints such as procurement contracts, workforce capacity, political considerations and community acceptability. The counterfactual (fixed spending) comparator is required to attribute improvements under optimized spending but is artificially unfavourable and does not represent a plausible policy trajectory. Other sources of uncertainty include the following: Spending and programme parameters are based on data available at the time of individual analyses and may not reflect current estimates. In particular, analyses should be revisited as pricing of long‐acting injectable PrEP evolves [36, 37]. Modelled populations and outcomes are informed by epidemiological and programmatic data which are subject to bias and often lack consistent disaggregation by key population, gender identity, Indigenous status, as well as sub‐populations who may be at higher risk of acquiring HIV, such as young men who have sex with men [38]. Finally, these models do not capture geographical heterogeneity in HIV epidemiology and service access, nor equity in intervention coverage, which should be considered in policy development and localized programme implementation.

## 5 Conclusions

This analysis shows that for five countries in the Latin America and Caribbean region, HIV transmission remains concentrated and shifting, and that substantial further reduction in the number of people acquiring HIV can be achieved through strategic reallocation of resources and targeted prevention for key populations. However, success will depend on strengthening surveillance, addressing structural barriers and protecting prevention programmes in a scarce financial landscape. Reductions in treatment costs can provide greater budget flexibility to resource‐focused services for key and marginalized populations, helping to close HIV prevention and care gaps and achieve more equitable epidemic gains.

## Author Contributions

The authors contributed to the manuscript in the following ways. Conceptualization: DtB, ALB, RM‐H and NS; Data curation: DtB, ALB, RM‐H, NW, KB, CYRH, RLN, BCM, RVSC, JPMC, SMNR and MPB; Formal analysis: DtB, ALB, RM‐H, NW, KB and NS; Methodology: DtB, ALB, RM‐H, KB and NS; Writing – original draft: DtB and ALB; Writing – review and editing: all authors. All authors read and approved the final manuscript.

## Funding

Individual country studies were funded through the support of the Global Fund to Fight AIDS, Tuberculosis and Malaria. No specific funding was received for the multi‐country analysis. The authors gratefully acknowledge the contribution to this work of the Victorian Operational Infrastructure Support Program received by the Burnet Institute. The Global Fund were involved in the interpretation of results and have co‐authored this manuscript.

## Conflicts of Interest

The authors have no conflicts of interest to declare.

## Supporting information




**Supporting Information File 1**: Supplementary information and results.
**Supplementary Appendix A**: Optima HIV model parameters.
**Supplementary Appendix B**: Country‐specific data inputs.
**Supplementary Appendix C**: Detailed results.

## Data Availability

Key data inputs are available in this manuscript and Supplementary Material. These were derived from a combination of sources in the public domain as well as data and reports provided by country‐partners. Optima HIV is a free and open‐source model and is available from GitHub (https://github.com/optimamodel/optima) with a user interface accessible from https://optimamodel.com/hiv/.
