# Supplementary material for: Optimizing HIV Spending Among Key Populations in Five Latin American and Caribbean Countries: A Mathematical Modelling Study
Source: J Int AIDS Soc. 2026 Jul 25;29(Suppl 3):e70171. doi: 10.1002/jia2.70171 (PMC13401690; doi:10.1002/jia2.70171)
Supplement: Supplementary file 1 — Supporting Information File 1: Supplementary information and results. Supplementary Appendix A: Optima HIV model parameters. Supplementary Appendix B: Country‐specific data inputs. Supplementary Appendix C: Detailed results. [file JIA2-29-e70171-s001.pdf]

Supplementary information and results

Optimizing HIV spending among key populations in five Latin American and Caribbean countries: a mathematical modelling study

Contents

Appendix A. Optima HIV model parameters 3
- Model parameters: constants 3
- Model parameters: setting specific and time-varying 5

Appendix B. Country-specific data inputs 7
- Epidemiological data inputs 7
- Intervention data inputs 10

Appendix C. Detailed results 16
- Key calibration figures 16
- Counterfactual (fixed spending) scenario 19
- Optimized spending allocation by country 21
- Projected epidemic impact of 100% spending optimized with uncertainty bounds 23
- Projected epidemic impact of 100% spending optimized (reduced ART costs) with uncertainty bounds 25
- Sensitivity analysis: Alternative optimization objectives 27

References 30

List of tables

Table A-1. Model parameters: transmissibility, disease progression and disutility weights. 3
Table A-2. Model parameters: treatment recovery and CD4 changes due to ART, and death rates. 4
Table A-3. Modeled setting-specific parameters in Optima HIV analyses and key sources 5
Table B-1. Most recent estimates of population size and HIV prevalence, Colombia 7
Table B-2. Most recent estimates of population size and HIV prevalence, Costa Rica 8
Table B-3. Most recent estimates of population size and HIV prevalence, Dominican Republic 8
Table B-4. Most recent estimates of population size and HIV prevalence, El Salvador 9
Table B-5. Most recent estimates of population size and HIV prevalence, Honduras 10
Table B-6. Modeled intervention description, unit cost per person per year, most recent coverage and maximum potential coverage in Colombia 11
Table B-7. Modeled intervention description, unit costs per person per year, most recent coverage and maximum potential coverage in Costa Rica 12
Table B-8. Modeled intervention description, unit costs per person per year, most recent coverage and maximum potential coverage in Dominican Republic 13
Table B-9. Modeled intervention description, unit costs per person per year, most recent coverage and maximum potential coverage in El Salvador 14
Table B-10. Modeled intervention description, unit costs per person per year, most recent coverage and maximum potential coverage in Honduras 15
Table C-1. Most recent and optimized spending allocation by country and intervention, 2024 US$ 21
Table C-2. Change in spending allocation by intervention and country with 100% spending optimized to minimize HIV-related deaths 27
Table C-3. Change in spending allocation by intervention and country with 100% spending optimized to minimize new HIV acquisitions 28
Table C-4. Change in spending allocation by intervention and country with 100% spending optimized to minimize disability-adjusted life years (DALYs) 29

List of figures

Figure C-1. Model calibration for each country to estimates of new HIV acquisitions, HIV-related deaths, total people living with HIV (PLHIV) and the HIV care cascade (specifically the total number of people on treatment). 16
Figure C-2. Model-projected incidence rates per 100 person-years from 2010–2030 by country and key population group under the counterfactual (fixed spending) scenario 19
Figure C-3. Number of new HIV acquisitions and HIV-related deaths, 2022-2030, under the counterfactual (fixed spending) and 100% spending optimized scenarios by country 23
Figure C-4. Number of new HIV acquisitions and HIV-related deaths, 2022-2030, under the counterfactual (fixed spending) and 100% spending optimized with reduced ART costs scenarios by country 25

Appendix A. Optima HIV model parameters

Model parameters: constants

This model is informed by the latest evidence on HIV transmission, disease progression, and the impact of HIV interventions on both. Table A1 lists all model assumptions with associated references found in the Optima HIV User Guide Volume VI Parameter Data Sources. Additional information on the structure of the Optima HIV model is available through Kerr (2015) [1].

Three different types of HIV transmission are modeled: transmission between sexual partners, transmission via sharing injecting equipment, and mother-to-child transmission. The input data associated with populations, sexual partnerships, injecting partnerships, and births are outlined in Table A-1 and Table A-2.

Table A-1. Model parameters: transmissibility, disease progression and disutility weights.

Interaction-related transmissibility (% per act)	
Insertive penile-vaginal intercourse	0.04%
Receptive penile-vaginal intercourse	0.08%
Insertive penile-anal intercourse	0.11%
Receptive penile-anal intercourse	1.38%
Intravenous injection	0.80%
Mother-to-child (breastfeeding)	36.70%
Mother-to-child (non-breastfeeding)	20.50%
Relative disease-related transmissibility	
Acute infection	5.60
CD4 (>500)	1.00
CD4 (500) to CD4 (350-500)	1.00
CD4 (200-350)	1.00
CD4 (50-200)	3.49
CD4 (<50)	7.17
Disease progression (average years to move)	
Acute to CD4 (>500)	0.24
CD4 (500) to CD4 (350-500)	0.95
CD4 (350-500) to CD4 (200-350)	3.00
CD4 (200-350) to CD4 (50-200)	3.74
CD4 (50-200) to CD4 (<50)	1.50
Changes in transmissibility (%)	
Condom use	95%
Circumcision	58%
Diagnosis behavior change	0%
STI cofactor increase	265%
Opioid substitution therapy	54%
PMTCT	90%
ARV-based pre-exposure prophylaxis (long-acting)	95%[1]
ARV-based pre-exposure prophylaxis (oral)	90%[1]
ARV-based post-exposure prophylaxis	73%
ART not achieving viral suppression	50%
ART achieving viral suppression	100%
Disutility weights	
Untreated HIV, acute	0.18
Untreated HIV, CD4 (>500)	0.01
Untreated HIV, CD4 (350-500)	0.03
Untreated HIV, CD4 (200-350)	0.08

Untreated HIV, CD4 (50-200)	0.29
Untreated HIV, CD4 (<50)	0.58
Treated HIV	0.08

1 Change in transmissibility uses a constant based on PrEP efficacy. This is modified on a country and population level according to model structure and the way that local coverage data are defined, PrEP modality, average continuation and adherence, and relative risk of those on PrEP through conversion to proportion of risk events that are covered and a population factor (see also Table A-3).
Source: Parameters were informed by multiple sources, reported in Optima HIV User Guide Volume VI Parameter Data Sources, and adapted based on Optima HIV model structure

Table A-2. Model parameters: treatment recovery and CD4 changes due to ART, and death rates.

Treatment recovery due to suppressive ART (average years to move)	
CD4 (350-500) to CD4 (>500)	2.20
CD4 (200-350) to CD4 (350-500)	1.42
CD4 (50-200) to CD4 (200-350)	2.14
CD4 (<50) to CD4 (50-200)	0.66
Time after initiating ART to achieve viral suppression (years)	0.20
**CD4 change due to non-suppressive ART (%/year)**	
CD4 (500) to CD4 (350-500)	3%
CD4 (350-500) to CD4 (>500)	15%
CD4 (350-500) to CD4 (200-350)	10%
CD4 (200-350) to CD4 (350-500)	5%
CD4 (200-350) to CD4 (50-200)	16%
CD4 (50-200) to CD4 (200-350)	12%
CD4 (50-200) to CD4 (<50)	9%
CD4 (<50) to CD4 (50-200)	11%
**Death rate (% HIV-related mortality per year)**	
Acute infection	0%
CD4 (>500)	0%
CD4 (350-500)	1%
CD4 (200-350)	1%
CD4 (50-200)	6%
CD4 (<50)	32%
Relative death rate on ART achieving viral suppression	23%
Relative death rate on ART not achieving viral suppression	49%

Source: Parameters were informed by multiple sources, reported in Optima HIV User Guide Volume VI Parameter Data Sources, and adapted based on Optima HIV model structure

Model parameters: setting specific and time-varying

Country-specific epidemiological, behavioral and programmatic data informing the Optima HIV model for participating countries were sourced from national records, surveys and other studies supplemented by expert advice from stakeholder consultations. Country-specific references are cited in individual country reports available via www.optimamodel.com/hiv and in Appendix B.

Table A-3. Modeled setting-specific parameters in Optima HIV analyses and key sources

Parameter	Source
Population size*	Age and gender stratified population sizes from the United Nations World Population Prospects 2024 (or latest year at time of work)[2] or country-preferred estimates.<br>Population sizes for key populations are estimated through population size estimation studies, census (populations in prisons), or expert opinion.
HIV prevalence by population groups*	HIV prevalence data values are used as the primary point of reference during calibration. Values are taken from a combination of primary research including survey data, where available, and expert opinion/assumptions where no data exists.
Other epidemiology*<br>▪ Percentage of people who die from non-HIV-related causes per year<br>▪ Prevalence of any ulcerative STIs<br>▪ Tuberculosis prevalence	Background mortality is taken from World Population Prospects, with supplementary comorbidity information from WHO reports for TB prevalence and behavioral surveys for STI prevalence.
Testing and treatment*<br>▪ Percentage of population tested for HIV in the last 12 months<br>▪ Probability of a person with CD4<200 being tested per year<br>▪ Number of people on treatment<br>▪ Proportion of exposure events covered by ARV-based pre-exposure prophylaxis (PrEP)<br>▪ Proportion of exposure events covered by ARV-based post-exposure prophylaxis<br>▪ Number of women on PMTCT (Option B/B+)<br>▪ Birth rate (births per woman per year)<br>▪ Percentage of HIV-positive women who breastfeed	The percentage of the population tested per year represents the likelihood that someone with an undiagnosed HIV infection will be diagnosed over the course of a year. As such inputs may be adjusted as part of calibration to match the proportion of new HIV acquisitions estimated to be diagnosed in each year, while maintaining trends in reported testing percentages. Sources include IBBS and household surveys.<br><br>PrEP person-coverage adjusted to proportion of exposure events covered according to the type of coverage data available, PrEP modality, average continuation, adherence, and relative risk of those on PrEP compared to those not on PrEP.
Optional indicators*<br>▪ Number of HIV tests per year<br>▪ Number of HIV diagnoses per year<br>▪ Modeled estimate of new HIV acquisitions per year<br>▪ Modeled estimate of HIV prevalence<br>▪ Modeled estimate of number of PLHIV<br>▪ Number of HIV-related deaths<br>▪ Number of people initiating ART each year<br>▪ PLHIV aware of their status (%)<br>▪ Diagnosed PLHIV in care (%)<br>▪ PLHIV in care on treatment (%)<br>▪ Pregnant women on PMTCT (%)<br>▪ People on ART with viral suppression (%)	Data entered in this section of the Optima HIV databook are not used by the model directly to generate output. These inputs allow comparison points to be entered from other reliable sources or models in order to ensure consistency. In this case Optima output were compared to Spectrum findings that have already been approved nationally through a consultative process.

Parameter	Source
Cascade*	
▪ Average time taken to be linked to care (years) (by population groups)<br>▪ Average time taken to be linked to care for people with CD4<200 (years)<br>▪ Percentage of people in care who are lost to follow-up per year (%/year)<br>▪ Percentage of people with CD4<200 lost to follow up (%/year)<br>▪ Percentage of people lost to follow-up who are returned to care per year (%/year)<br>▪ Viral load monitoring (number/year)<br>▪ Proportion of those with identified viral load failure who are provided with effective adherence support or a successful new regimen (%/year)<br>▪ Treatment failure rate	Cascade parameters informed by programmatic data and expert opinion.
Sexual behaviors*	
▪ Average number of acts with regular partners per person per year<br>▪ Average number of acts with casual partners per person per year<br>▪ Average number of acts with transactional partners per person per year<br>▪ Percentage of people who used a condom at last act with regular partners<br>▪ Percentage of people who used a condom at last act with casual partners<br>▪ Percentage of people who used a condom at last act with transactional partners<br>▪ Percentage of males who have been traditionally circumcised<br>▪ Number of voluntary medical male circumcisions	Sources for sexual behavior include IBBS and household surveys. Number of voluntary medical male circumcisions informed by programmatic data.
Injecting behaviors*	
▪ Average number of injections per person per year<br>▪ Percentage of people who receptively shared a needle/syringe at last injection<br>▪ Number of people who inject drugs who are on opioid substitution therapy	Sources for injecting behavior include IBBS and other survey data.
Partnerships and transitions	
▪ Interactions between regular partners<br>▪ Interactions between casual partners<br>▪ Interactions between transactional partners<br>▪ Interactions between people who inject drugs<br>▪ Birth<br>▪ Aging<br>▪ Risk-related population transitions (average number of years before movement)	Informed by population definitions and behavioral survey data. Aging rates based on demographics in United Nations World Population Prospects 2022 (or latest year at time of work).<br>Risk transitions account for temporal behaviors and risks as well as overlap between population groups.
Migration*	
▪ Percentage of people who emigrate per year<br>▪ Number of people who immigrate into population per year<br>▪ HIV prevalence of immigrants into population per year<br>▪ Proportion of people living with HIV who immigrate who are diagnosed prior to arrival	Optionally included depending on significance to epidemic and available data. Sources for numbers of emigrants and immigrations based on United Nations World Population Prospects or national reports (e.g. Migración Colombia). Estimates for HIV prevalence and diagnosis among migrants informed by programmatic or survey data.<br>Migration flows modeled in Colombia and Costa Rica.

ART, antiretroviral treatment; IBBS, integrated biological behavioral surveillance surveys; PLHIV, people living with HIV; PMTCT, prevention of mother-to-child transmission (vertical transmission); STI, sexually transmitted infection
*Values can be defined annually for each year

Appendix B. Country-specific data inputs

Epidemiological data inputs

Unless an alternative source was nominated by country representatives, overall population size was derived from United Nations Population Division World Population Prospects. Population sizes for key and priority populations were derived from the most recent estimates from survey data, expert consensus or population census and projected for subsequent years based on a fixed proportion of the population denominator unless otherwise indicated.

Table B-1. Most recent estimates of population size and HIV prevalence, Colombia

	Population size		HIV prevalence	
Population (age group modeled)	Latest estimate (% of population)	Year of estimate; Source	Latest estimate	Source
Female sex workers (18-49)	248,000 (2.0% F18-49)	2021; 2023 KPAtlas	0.8%	Based on program data (GAM 2022). Previous estimate was 1.1% based on 2012 IBBS.
Clients of female sex workers (18-49)	2,480,000 (21.0% of M18-49) [1]	2021; Assumed 10 times population of FSW	N/A	
Men who have sex with men (18+)	578,708 (3.2% M18+)	2023; Enterritorio. Informe GAM - ONUSIDA: Prevencion VIH Colombia 2024: Ministerio de Salud y Protección Social; 2024	20.4%	2019 IBBS [3]
Transgender women (18+)	12,317 (0.07% M18+)	2023; Enterritorio. Informe GAM - ONUSIDA: Prevencion VIH Colombia 2024: Ministerio de Salud y Protección Social; 2024	23.4%	2019 IBBS [3]
People who inject drugs (18-49)	15,500 (0.13% M18-49)	2023; 2025 KPAtlas	11.1%	2021 IBBS [4,5]
People deprived of liberty (males 18-49)	101,622 (0.08% M18-49)	2023; Enterritorio. Informe GAM- ONUSIDA: Prevencion VIH Colombia 2024: Ministerio de Salud y Protección Social; 2024	0.83%	GAM Online Reporting Tool: Colombia 2022
Men experiencing homelessness (males 18-49)	29,864 (0.25% M18-49)	2021; Censo de Habitantes de la Calle [6]	4.0%[1]	2015 IBBS [7]
Women experiencing homelessness (females 18-49)	4,227 (0.03% M18-49)		7.6%[1]	
Venezuelan migrants (males 18+)	955,147 (5.2% M18+)[2]	2024; Migración Colombia [8]	1.6%	2022 IBBS (Venezuelans living in Colombia) [9]
Venezuelan migrants (females 18+)	1,063,686 (5.4% F18+)[2]		0.6%	
Total population	52,117,067	2023; Departamento Administrativo Nacional de Estadística (DANE) 2025 [10]	N/A	

FSW, female sex workers; GAM, Global AIDS Monitoring; IBBS, integrated biological behavioral survey [estudio de comportamiento sexual y prevalencia de infección por VIH]; KPAtlas, Key Population Atlas

[1] Low confidence in estimate (estimate more than five years old at time of analysis or based on assumption, with no better estimates identified by country teams);

[2] High uncertainty in future projections of migrant population size. Modeled population sizes assume fixed number of migrants from 2024 onwards.

Table B-2. Most recent estimates of population size and HIV prevalence, Costa Rica

	Population size		HIV prevalence	
Population (age group modeled)	Latest estimate (% of population)	Year of estimate; Source	Latest estimate	Source
Female sex workers (15-49)	3,020 (0.2% F14-49)	2018; Country-provided data	1.3%	2018 IBBS [11]
Clients of female sex workers (15-49)	30,200 (2.3% M15-49) [1]	2018; Assumed 10 times population of FSW	NA	
Men who have sex with men (15-49)	10,543 (0.8% M15-49)	2019; Informe de desarrollo de cascadas de prevencion y atencion, 2019 Dra Rodriguez Artiga Sep 2020	15.4%	2018 IBBS [11]
Transgender women (15-49)	416 (0.3% M15-49)	2018; 2018 IBBS [11]	23.0%	2018 IBBS [11]
People in prison, male (15-49)	15,609 (1.2% M15-49)	2017; Ministerio de Justicia y Paz. Anuario 2017	0.3%	KPAtlas (UNAIDS 2021). Source: Ministerio de Justicia y Paz (2020)
Total population	4,876,273	2019; World Population Prospects 2019	NA	

FSW, female sex workers; IBBS, integrated biological behavioral survey [Encuesta de comportamiento sexual y prevalencia de VIH e ITS y estimación del tamaño de poblaciones clave]; KPAtlas, Key Population Atlas; NA, not available.
[1] Low confidence in estimate (estimate more than five years old at time of analysis or based on assumption, with no better estimates identified by country teams)

Table B-3. Most recent estimates of population size and HIV prevalence, Dominican Republic

	Population size		HIV prevalence	
Population (age group modeled)	Latest estimate (% of population)	Year of estimate; Source	Latest estimate	Source
Female sex workers (15-49)	102,908 (3.8% F15-49)	2019; Average of:<br>1) CONAVIHSIDA / ASESAI 2019 (=3.7% F15-49)[12]<br>2) (2011) KPAtlas (=3.9% F15-49)	3.3%	2021 IBBS (Preliminary results; median) [13]
Clients of female sex workers (15-49)	350,060 (13% M15-49)	2019; Average of:<br>1) ENDESA 2013, paid for sex in lifetime (20.8%)<br>2) ENDESA 2013, paid for sex in past year (5.1%) [14]	1.9%[1]	Based on men reporting paying for sex in the past 12 months, ENDESA 2013 [14]
Men who have sex with men (15-49)	125,942 (4.7% M15-49)	2019; Average of:<br>1) CONAVIHSIDA / ASESAI 2019 (=4.4% M15-59) [12]<br>2) (2017) KPAtlas (=4.9% M15-49)	7.0%	2021 IBBS (Preliminary results; median) [13]
Transgender women (15-49)	7,299 (0.27% M15-49)	2019; CONAVIHSIDA / ASESAI 2019 (=0.27% M15-59) [12]	32.0%	2021 IBBS (Preliminary results; based on Santo Domingo only) [13]
People in prison, male (15-49)	23,118 (0.85% M15-49)	2020; Average of:<br>1) Censo de Población PPL (0.82%) [12]<br>2) (2020) PEN 2021-2024 [15]	4.7%	2021 IBBS (Preliminary results) [13]
People who use drugs, male (15-49)	58,835 (2.3% M15-49) [1]	2013; ENDESA 2013 [14]	3.2%[1]	2012 IBBS [16]
Haitian migrant females (15-49)	185,390 (6.9% F15-49)	2017; ENI-2017 [17]	4.0%	2018 IBBS (not stratified by sex) [18]
Haitian migrant males (15-49)	308,614 (11.6% M15-49)		4.0%	2018 IBBS (not stratified by sex) [18]
Total population	10,448,499	2020; Oficina Nacional de Estadística [National Statistical Office] (ONE)	NA	

ASESAI, Apoyo técnico: Asesoría y Servicios en Salud Internacional [Technical Support: International Health Advisory and Services]; CONAVIHSIDA, Consejo Nacional para el VIH y el SIDA [National Council for HIV and AIDS]; IBBS, integrated biological behavioral survey [Encuesta de Vigilancia de Comportamiento con Vinculación Serológica]; ENDESA, la Encuesta Demográfica y de Salud [Demographic and Health Survey]; ENI, Encuesta Nacional de Inmigrantes [National survey of immigrants]; NA, not available; WPP, World Population Prospects

[1] Low confidence in estimate (estimate more than five years old at time of analysis or based on assumption, with no better estimates identified by country teams)

Table B-4. Most recent estimates of population size and HIV prevalence, El Salvador

	Population size		HIV prevalence	
Population (age group modeled)	Latest estimate (% of population)	Year of estimate; Source	Latest estimate	Source
Female sex workers (15-49)	54,140 (3.0% F15-49)	2016 ; KPAtlas (UNAIDS 2021)	1.2%	Sentinel surveillance 2019 via KPAtlas (UNAIDS 2021)
Clients of female sex workers (15-49)	541,400 (35% M15-49) [1]	2016; Assumed 10 times population of FSW	NA	
Men who have sex with men (15-49)	44,972 (2.9% M15-49)	2016 ; KPAtlas (UNAIDS 2021)	7.8%	Sentinel surveillance 2019 via KPAtlas (UNAIDS 2021)
Transgender women (15-49)	1,835 (0.12% M15-49) [1]	2014 ; KPAtlas (UNAIDS 2021)	15.3%	Sentinel surveillance 2018 via KPAtlas (UNAIDS 2021)
People in prison, male (15-49)	34,401 (2.2% M15-49)	2020; World Prison Brief (2020) and KPAtlas (UNAIDS 2021)	0.1%	KPAtlas (UNAIDS 2021) – Tests conducted in prisons and other closed settings
Total population	6,486,201	2020; 2019 World Population Prospects	NA	

FSW, female sex workers; KPAtlas, Key Population Atlas; NA, not available.

[1] Low confidence in estimate (estimate more than five years old at time of analysis or based on assumption, with no better estimates identified by country teams)

Table B-5. Most recent estimates of population size and HIV prevalence, Honduras

	Population size		HIV prevalence	
Population (age group modeled)	Latest estimate (% of population)	Year of estimate; Source	Latest estimate	Source
Female sex workers (15-49)	28,256 (1.1% F15-49)	2019; Country-provided data derived from Cuadro resumen de la estimación del tamaño de las poblaciones clave en Honduras según informe de estudio (2016)	2.2%	Country-provided data (Resumen de Subpoblaciones, de Estimaciones 2019 Honduras Spectrum)
Clients of female sex workers (15-49)	282,560 (11% M15-49)[1]	2019; Assumed 10 times population of FSW	NA	
Men who have sex with men (15-49)	44,719 (1.4%)	2019; Country-provided data derived from Cuadro resumen de la estimación del tamaño de las poblaciones clave en Honduras según informe de estudio (2016)	10.8%	Country-provided data (Resumen de Subpoblaciones, de Estimaciones 2019 Honduras Spectrum)
Transgender women (15-49)	2,621 (0.1% M15-49)	2019; Country-provided data derived from Cuadro resumen de la estimación del tamaño de las poblaciones clave en Honduras según informe de estudio (2016) and applied % to M15-49 (2019)	11.4%	Country-provided data (Resumen de Subpoblaciones, de Estimaciones 2019 Honduras Spectrum)
People in prison, male (15-49)	21,708 (0.8% M15-49)	2020; Instituto Nacional Penitenciario. Población Penal, 31 December 2020	0.6%	Country-provided data (Resumen de Subpoblaciones, de Estimaciones 2019 Honduras Spectrum)
Garifunas Indigenous population, males (15-49)	75,226 (3.6% M15-49)[1]	2010; Based on an estimate of 300,000 people (all ages) adjusted to 15-49 assuming same age distribution as total population.	4.4%[1]	2012 IBBS (ECVC) [19]
Garifunas Indigenous population, females (15-49)	77,984 (3.7% F15-49)[1]		4.6%[1]	
Total population	9,746,115	2019; World Population Prospects 2019	NA	

IBBS, integrated biological behavioral survey [ECVC, Encuesta Centroamericana de Vigilancia Comportamientos sexual y prevalencia de VIH/ITS de población clave]; FSW, female sex workers. NA, not available.
[1] Low confidence in estimate (estimate more than five years old at time of analysis or based on assumption, with no better estimates identified by country teams).

Intervention data inputs

Table B-6 to Table B-10 outline program definitions by country, including a description of what is included in each intervention, unit cost (cost per person per year), most recent coverage estimate as a proportion of the population reached, and maximum potential coverage of intervention among target population(s) accounting for geographical, social and implementation constraints in accessibility and uptake.

Table B-6. Modeled intervention description, unit cost per person per year, most recent coverage and maximum potential coverage in Colombia

	Colombia			
Intervention	Description	Unit cost[1]	Latest coverage	Maximum coverage[2]
ART	Includes costs related to antiretroviral (ARV) medication, consultations and follow-up, clinical examinations, HIV-specific monitoring, and social support for adherence and retention [20]. Excludes costs related to prevention, testing, and treatment of co-infections and opportunistic infections. A weighted average cost of treatment for adults and children was estimated, and it was assumed that the cost was the same for the treatment of all population groups based on feedback from national stakeholders.	$688.12	82%[3]	100.0%
PMTCT	Includes ARVs, consultations and follow-up with healthcare professionals, HIV-specific clinical tests and monitoring, and nutritional support. The cost of antenatal screening is not included.	$888.58	92%[4]	99.0%
HIV testing for the general population	Based on facility-based testing among non-key population groups, migrants and clients of FSW. Unit cost is based on the basic supplies for third- and fourth-generation rapid HIV tests, plus the estimated per-person testing costs for key populations (to ensure comparability with testing costs for key populations, which includes operational costs and human resources).	$10.75	3.7%	33.0%
Condom distribution/promotion among the general population	Based on a total of 4,642,011 condoms distributed through healthcare facilities, which are assumed to be primarily used by the general population. It is assumed that 9 condoms are distributed per prevention kit, twice a year. Commodity-cost only.	$0.65	0.7%	18.0%
PrEP (oral)	Available only to men who have sex with men and transgender women. Includes the annual cost of medication, required tests and follow-ups, and health consultations [21].	$189.84	1.0%[5]	53.3%[5]
PrEP (long-acting)	Prospective intervention. Estimated annual cost for medication based on estimated cost for injectable cabotegravir in 2025 in low- and middle-income countries [22], and frequency of follow up adjusted based on a schedule of two injections at initiation one month apart, and every two months thereafter [21].	$260.03	0.0%[5]	53.3%[5]
HIV self-testing	Program cost estimates provided by the country based on one self-test kit per person reached.	$3.02	0.0%	45.0%
HIV prevention and testing services for MSM	Includes the cost of basic supplies (condoms, lubricants, third- and fourth-generation rapid HIV tests), personnel costs, and transportation. It assumes that beneficiaries receive two prevention kits per year.	$20.79	12.5%	85.0%
HIV prevention and testing services for transgender women		$20.79	38.0%	85.0%
HIV prevention and testing services for FSW		$20.79	7.1%	85.0%
HIV prevention and testing services for PWID	As above, plus the cost of the needle, syringes, and tourniquet included in the prevention kit.	$30.01	28.8%	85.0%
HIV prevention and testing services for migrants	Based on the distribution of HIV/syphilis prevention and dual-test kits through AFA. The weighted cost for the prevention component accounts for one kit per person on AFA prevention days, 10 kits per person for people living with HIV, and six kits per person for AHF [23].	$26.08	3.2%	15.0%
HIV prevention and testing services for people in prisons	Prevention and HIV testing for people deprived of liberty (people in prisons and other closed settings). Assumed same cost as for testing among the general population.	$10.75	2.5%	85.0%

AFA, AID for AIDS; AHF, AIDS Healthcare Foundation; ANC, antenatal care; ART, antiretroviral treatment; ARV, antiretroviral; FSW, female sex workers; HTS, HIV testing services; MSM, men who have sex with men; PEP, post-exposure prophylaxis; PMTCT, prevention of mother-to-child transmission; PrEP, pre-exposure prophylaxis; PWID, people who inject drugs; TGW, transgender women.
[1] Unit cost calculated per person per year
[2] Maximum potential coverage of interventions among target population(s) accounting for geographical, social and implementation constraints in accessibility and uptake;
[3] ART coverage based on estimated proportion of diagnosed people living with HIV on ART in modeled outputs (2024);
[4] PMTCT coverage based on modeled output of proportion of pregnant women living with HIV receiving PMTCT (2024);
[5] PrEP coverage based on estimated proportion of risk covered at a population level accounting for the relative risk of those on PrEP compared to those not on PrEP.
Source: Program data and existing costing studies

Table B-7. Modeled intervention description, unit costs per person per year, most recent coverage and maximum potential coverage in Costa Rica

	Costa Rica			
Intervention	Description	Unit cost[1]	Latest coverage	Maximum coverage[2]
ART	Antiretroviral treatment	$526.78	87%[3]	100.0%
PMTCT	Antiretrovirals for prevention of vertical transmission	$1,027.58	56%[4]	100.0%
HIV testing services	HIV testing and counselling for all populations	$29.34	1.1%	80.0%
Condom distribution/promotion among the general population	Condom distribution for adults outside of key populations	$9.89	7.0%	75.0%
PrEP (oral)	PrEP reaching MSM and transgender women	$468.22	6.2%[5]	30.0%[5]
HIV prevention services for MSM	Based on HIV prevention (condoms and social and behavior change communication); excludes HIV testing	$77.72	28.0%	75.0%
HIV prevention services for transgender women	Based on HIV prevention (condoms and social and behavior change communication); excludes HIV testing	$444.91	56.1%	70.0%
HIV prevention services for FSW	Based on HIV prevention (condoms and social and behavior change communication); excludes HIV testing	$32.39	84.9%	85.0%
HIV prevention services for people in prisons	Prevention and HIV testing for people deprived of liberty (people in prisons and other closed settings)	$7.79	45.5%	75.0%

ART, antiretroviral treatment; FSW, female sex workers; MSM, men who have sex with men; PMTCT, prevention of mother-to-child transmission; PrEP, pre-exposure prophylaxis.
[1] Unit cost calculated per person per year
[2] Maximum potential coverage of interventions among target population(s) accounting for geographical, social and implementation constraints in accessibility and uptake;
[3] ART coverage based on estimated proportion of diagnosed people living with HIV on ART in modeled outputs (2024);
[4] PMTCT coverage based on modeled output of proportion of pregnant women living with HIV receiving PMTCT (2024);
[5] PrEP coverage based on estimated proportion of risk covered at a population level accounting for the relative risk of those on PrEP compared to those not on PrEP.
Source: Unit cost based on MEGAS 2019 (spending/coverage) and country-provided cost data.

Table B-8. Modeled intervention description, unit costs per person per year, most recent coverage and maximum potential coverage in Dominican Republic

	Dominican Republic			
Intervention	Description	Unit cost[1]	Latest coverage	Maximum coverage[2]
ART	Includes ARVs and implementation costs for treatment and PMTCT.	$448.26	84%[3]	95.0%
PMTCT	Includes safe infant breastfeeding practices and other PMTCT activities not specified, excluding ARVs and antenatal testing.	$315.78	91%[4]	99.5%
Treatment support programs	Incorporates differentiated package of adherence treatment retention support services, including nutritional packages, transportation support, tele-adherence, and home visits. Modeled to reduce loss-to-follow up.	$69.96	51.7%	70.0%
HIV testing for the general population	Represents general HIV testing services delivered through Servicio Nacional de Salud to the general population as well as antenatal screening, facility-based testing for migrants, and testing reaching clients of FSW and people who use drugs.	$7.02	14.8%	95.0%
Condom distribution/promotion among the general population	Differentiated by program type.			
Free	Based on provision of free condoms for HIV prevention and social and behavior change communication (SBCC), excluding key populations	$0.33	8.0%	90.0%
Social marketing	Social marketing of condoms for HIV prevention, excluding key populations	$0.59	19.5%	90.0%
PrEP (oral)	Population-stratified PrEP implementation, excluding PrEP specifically for serodiscordant couples, for MSM and transgender women, female sex workers, and Haitian migrants	$154.74	0.4%[5]	15.0%[5]
HIV prevention services for MSM	MSM-focused HIV prevention services including condom distribution and SBCC	$6.16	61.8%	95.0%
HIV testing services for MSM	MSM-focused HIV testing services	$10.54	61.0%	95.0%
HIV prevention services for transgender women	Transgender women-focused HIV prevention services including condom distribution and SBCC	$14.68	61.4%	95.0%
HIV testing services for transgender women	Transgender women-focused HIV testing services	$18.75	62.0%	95.0%
HIV prevention services for FSW	FSW-focused HIV prevention services including condom distribution and SBCC	$8.13	61.4%	95.0%
HIV testing services for FSW	FSW-focused HIV testing services	$10.31	65.9%	95.0%
HIV prevention services for migrants	Migrant-focused HIV prevention services including condom distribution and SBCC	$3.37	42.1%	70.0%
HIV testing services for migrants	Outreach HIV testing focused on Haitian migrants (excludes HIV tests conducted through SNS)	$7.82	17.4%	70.0%
HIV prevention and testing services for people in prisons	Combined HIV prevention and testing services for people in prisons	$11.43	0.5%	95.0%

ARV, antiretroviral; FSW, female sex workers; MSM, men who have sex with men; PMTCT, prevention of mother-to-child transmission; PrEP, pre-exposure prophylaxis; SBCC, social and behavior change communication; SNS, Servicio Nacional de Salud.

[1] Unit cost calculated per person per year

[2] Maximum potential coverage of interventions among target population(s) accounting for geographical, social and implementation constraints in accessibility and uptake;

[3] ART coverage based on estimated proportion of diagnosed people living with HIV on ART in modeled outputs (2024);

[4] PMTCT coverage based on modeled output of proportion of pregnant women living with HIV receiving PMTCT (2024);
[5] PrEP coverage based on estimated proportion of risk covered at a population level accounting for the relative risk of those on PrEP compared to those not on PrEP.
Source: 2020 MEGAS. Total spending converted to unit cost based on 2020 coverage.

Table B-9. Modeled intervention description, unit costs per person per year, most recent coverage and maximum potential coverage in El Salvador

	El Salvador			
Intervention	Description	Unit cost[1]	Latest coverage	Maximum coverage[2]
ART	Antiretroviral treatment	$306.12	68%[3]	100%
PMTCT	Antiretrovirals for prevention of vertical transmission	$412.02	77%[4]	100%
HIV testing for the general population	Facility-based testing for children and adults outside of defined key populations	$9.25	6%	75%
Condom distribution/promotion among the general population	Condom promotion and distribution for people outside of identified key populations, including clients of FSW. Condom programs were evaluated separately by funding source (international, public, private[5]), as requested by national collaborators (equivalent unit cost for all).	$2.45	21.2%	85%
Post-exposure prophylaxis	Post-exposure prophylaxis (PEP) reaching all adult populations	$7650.28	0.01%	64.9%
HIV prevention and testing services for MSM	Condom-based HIV prevention and testing services for MSM	$58.87	41%	80%
HIV prevention and testing services for transgender women	Condom-based HIV prevention and testing services for transgender women	$163.43	46%	75%
HIV prevention and testing services for FSW	Condom-based HIV prevention and testing services for FSW	$59.72	15%	80%
HIV prevention and testing services for people in prisons	Condom-based HIV prevention and testing services for people in prisons	$6.18	65%	65%

ART, antiretroviral treatment; FSW, female sex workers; MSM, men who have sex with men; PMTCT, prevention of mother-to-child transmission.
[1] Unit cost calculated per person per year
[2] Maximum potential coverage of interventions among target population(s) accounting for geographical, social and implementation constraints in accessibility and uptake;
[3] ART coverage based on estimated proportion of diagnosed people living with HIV on ART in modeled outputs (2024);
[4] PMTCT coverage based on modeled output of proportion of pregnant women living with HIV receiving PMTCT (2024);
[5] Spending for private sale of condoms assumed to be fixed and not part of the optimizable budget, thus private spending has been excluded from total HIV spending as part of this multi-country analysis.
Source: GAM 2020 (total spending divided by coverage) or regional unit cost.

Table B-10. Modeled intervention description, unit costs per person per year, most recent coverage and maximum potential coverage in Honduras

	Honduras			
Intervention	Description	Unit cost[1]	Latest coverage	Maximum coverage[2]
ART	Antiretroviral treatment	$1,490.30	78%[3]	100.0%
PMTCT	Antiretrovirals for prevention of vertical transmission	$12,218.68	35%[4]	100.0%
HIV testing for the general population	Facility-based HIV testing outside of modeled key populations	$13.61	2.3%	75.0%
Condom distribution/promotion among the general population	Condom distribution and social behavior change communication. Condom programs were evaluated separately by funding source, as requested by national collaborators.			
International		$6.42	1.1%	85.0%
Private[5]		$207.75[5]	0.6%	85.0%
Public		$28.36	2.0%	85.0%
HIV prevention and testing services for MSM	Condom-based HIV prevention and testing services for MSM	$104.54	25.0%	85.0%
HIV prevention and testing services for transgender women	Condom-based HIV prevention and testing services for transgender women	$240.74	21.0%	90.0%
HIV prevention and testing services for FSW	Condom-based HIV prevention and testing services for FSW	$302.51	9.5%	85.0%
HIV prevention and testing services for Indigenous populations	HIV prevention services, includes condom distribution and social and behavior change communication, and testing services for Garífunas people	$149.63	1.8%	75.0%
HIV prevention and testing services for people in prisons		$6.21	17.4%	75.0%

ART, antiretroviral treatment; FSW, female sex workers; MSM, men who have sex with men; PMTCT, prevention of mother-to-child transmission.
[1] Unit cost calculated per person per year
[2] Maximum potential coverage of interventions among target population(s) accounting for geographical, social and implementation constraints in accessibility and uptake;
[3] ART coverage based on estimated proportion of diagnosed people living with HIV on ART in modeled outputs (2024);
[4] PMTCT coverage based on modeled output of proportion of pregnant women living with HIV receiving PMTCT (2024);
[5] Spending for private sale of condoms assumed to be fixed and not part of the optimizable budget, thus private spending has been excluded from total HIV spending as part of this multi-country analysis.
Source: Country-provided data and MEGAS 2019

Appendix C. Detailed results

Key calibration figures

Figure C-1. Model calibration for each country to estimates of new HIV acquisitions, HIV-related deaths, total people living with HIV (PLHIV) and the HIV care cascade (specifically the total number of people on treatment).

Colombia | Main calibration source: Spectrum 2025

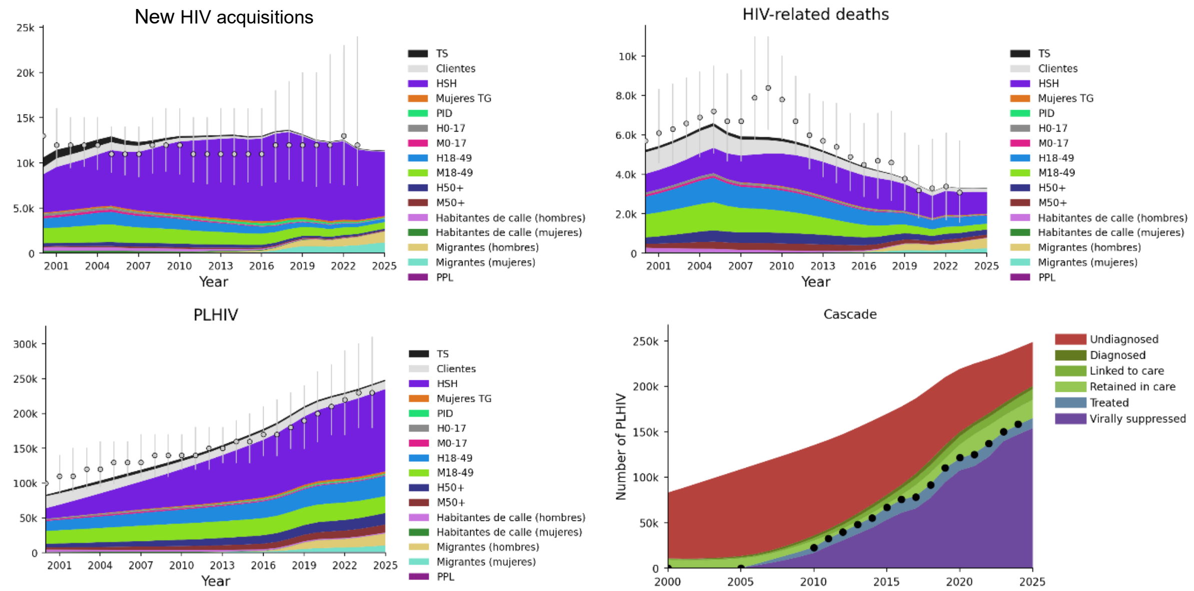

Costa Rica | Main calibration source: Spectrum 2025

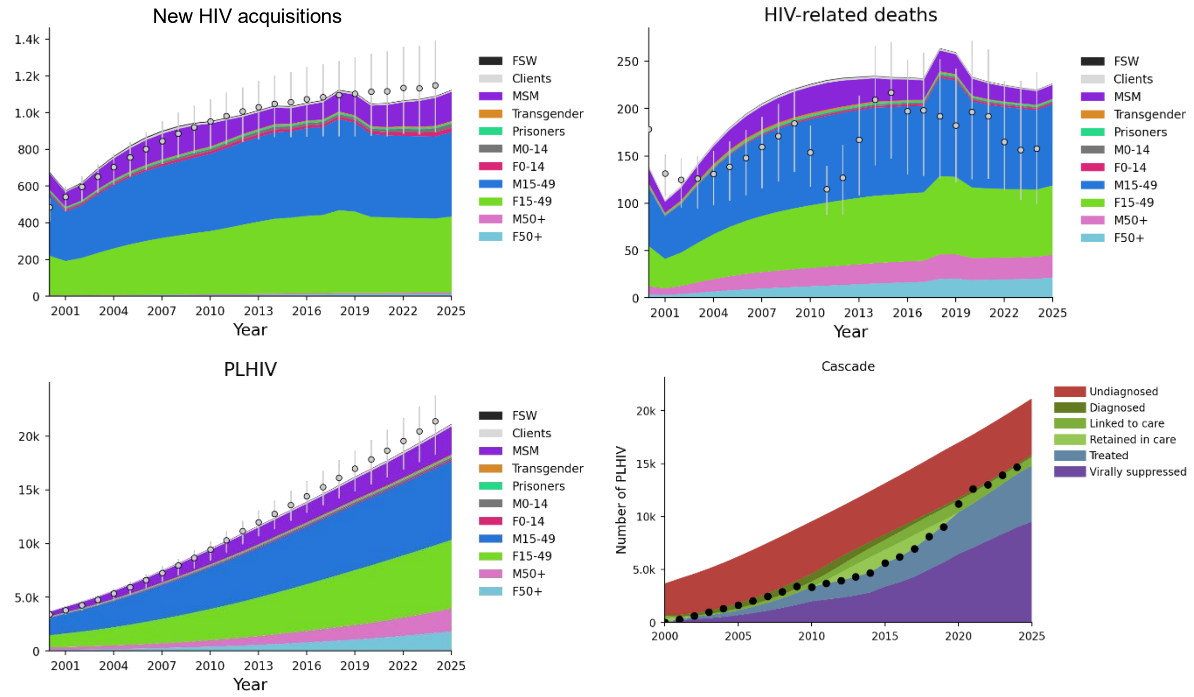

Dominican Republic

Main calibration source: Spectrum 2019 via MISPAS

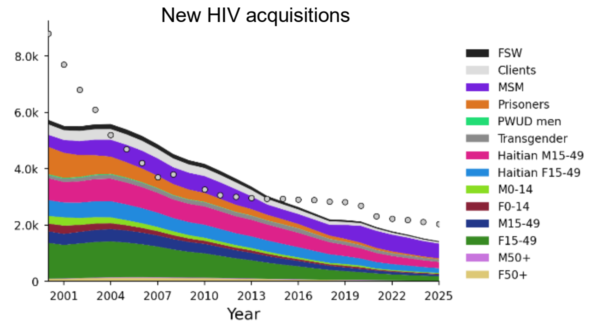

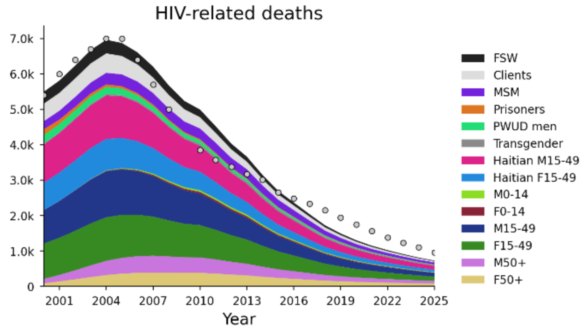

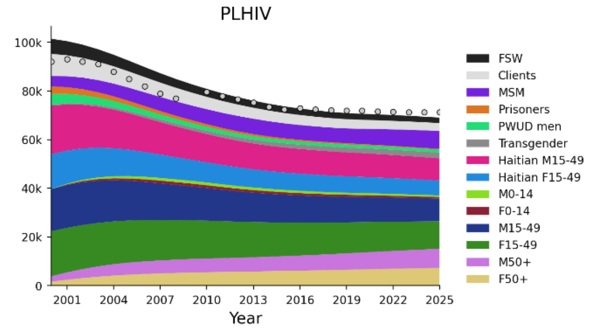

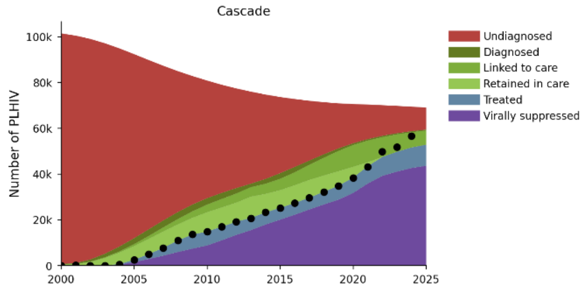

El Salvador

Main calibration source: SUMEVE, Spectrum 2020

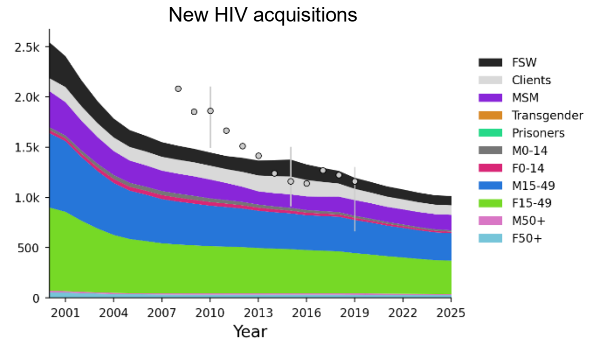

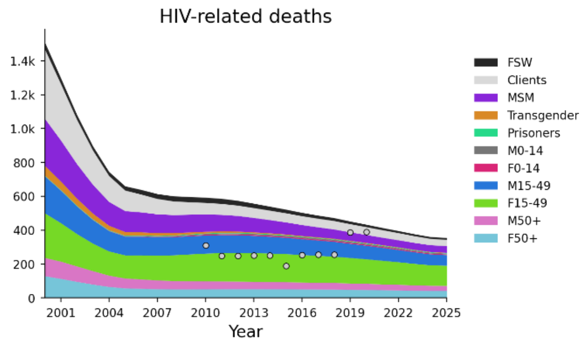

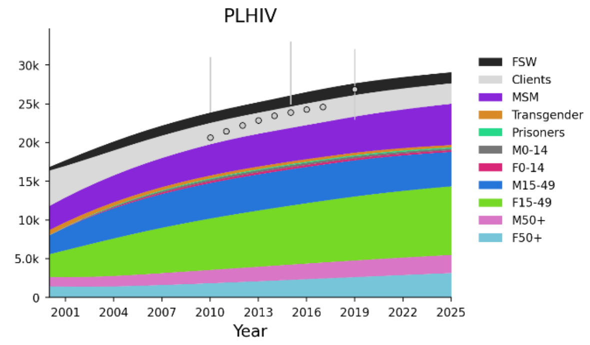

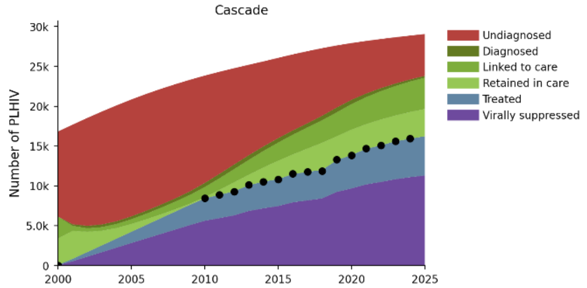

Honduras

Main calibration source: Spectrum 2020

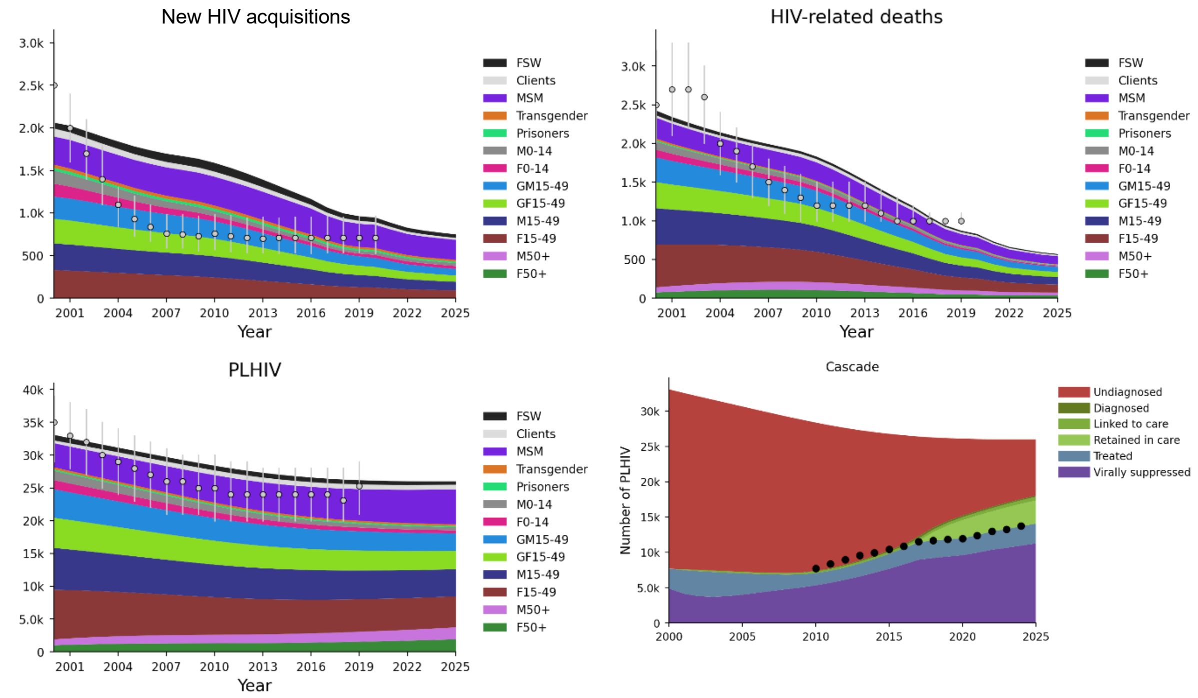

Counterfactual (fixed spending) scenario

Figure C-2. Model-projected incidence rates per 100 person-years from 2010–2030 by country and key population group under the counterfactual (fixed spending) scenario

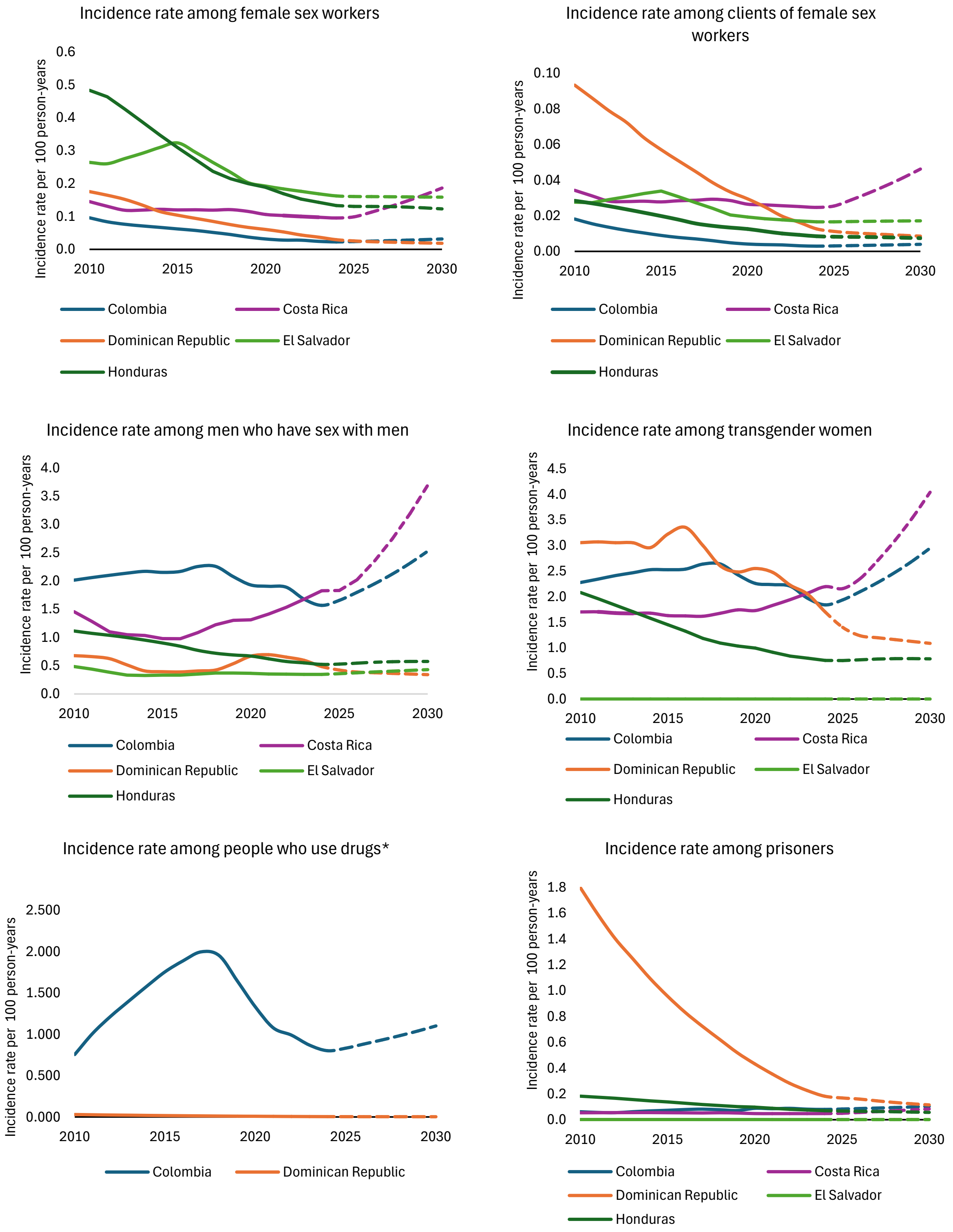

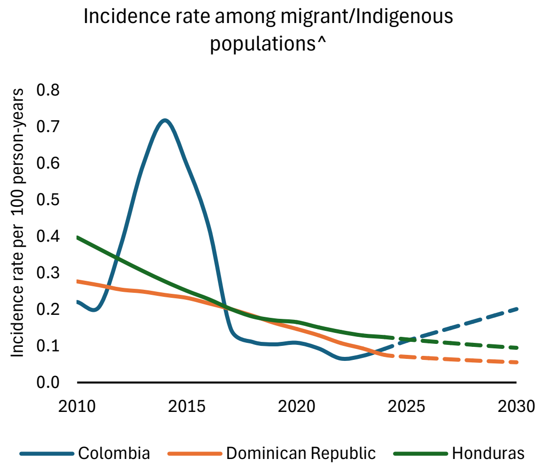

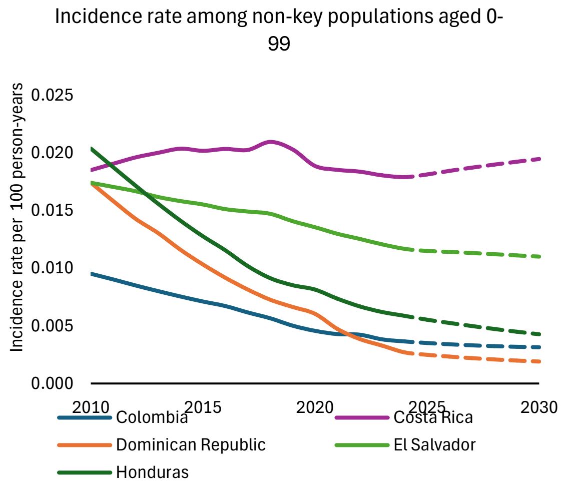

* Based on people who inject drugs in Colombia and people who use drugs through injecting and non-injecting routes in Dominican Republic

^ Migrant and Indigenous populations considers Venezuelan migrants in Colombia, Haitian migrants in Dominican Republic, and Garifuna population in Honduras.

Solid line represents historical trends calibrated to HIV prevalence trends and overall new HIV acquisitions from Spectrum estimates given treatment data up to 2024. Dashed line designates beginning of model projections (2025).

Optimized spending allocation by country

Table C-1. Most recent and optimized spending allocation by country and intervention, 2024 US$

Country	Colombia	Costa Rica	Dominican Republic	El Salvador	Honduras
**Most recent spending**					
ART	$108,473,384	$7,715,793	$25,393,926	$4,887,536	$20,472,262
PMTCT	$799,724	$36,993	$488,518	$35,434	$1,515,116
Treatment support programs	$0	$0	$1,854,477	$0	$0
HIV testing for the general population	$14,953,457	$1,359,147	$8,175,538	$3,583,700	$2,255,101
Condom distribution/promotion among the general population[1]	$169,322	$2,879,658	$1,038,791	$773,137	$4,527,111
PrEP[2]	$1,823,588	$325,880	$228,121	$3,870,804	$0
HIV self-testing	$42	$0	$0	$0	$0
HIV services for MSM[3]	$1,534,740	$246,581	$1,930,068	$1,254,504	$1,365,750
HIV services for transgender women[3]	$99,036	$147,263	$226,150	$172,755	$162,013
HIV services for FSW[3]	$379,837	$308,577	$1,541,988	$518,000	$909,052
HIV services for PWID[3]	$136,245	$0	$0	$0	$0
HIV services for migrants or Indigenous populations[3]	$1,685,583	$0	$1,466,471	$0	$620,072
HIV services for people in prisons[3]	$12,527	$66,243	$1,280	$417,231	$27,150
Total	$130,067,484	$13,086,135	$42,345,328	$15,513,102	$31,853,627
**Optimized spending**					
ART	$118,870,572	$9,373,550	$26,709,237	$6,622,837	$25,405,387
PMTCT	$799,724	$257,624	$488,518	$35,434	$1,515,116
Treatment support programs	$0	$0	$2,965,877	$0	$0
HIV testing for the general population	$7,476,728	$679,573	$4,087,769	$3,561,125	$1,127,551
Condom distribution/promotion among the general population[1]	$84,661	$1,439,829	$519,396	$386,568	$2,263,555
PrEP[2]	$911,794	$951,227	$1,199,930	$1,935,402	$0
HIV self-testing	$21	$0	$0	$0	$0
HIV services for MSM[3]	$767,370	$123,290	$2,923,683	$1,699,911	$682,875
HIV services for transgender women[3]	$49,518	$73,632	$383,431	$86,378	$81,006
HIV services for FSW[3]	$189,919	$154,288	$1,110,315	$976,831	$454,526
HIV services for PWID[3]	$68,122	$0	$0	$0	$0
HIV services for migrants or Indigenous populations[3]	$842,792	$0	$1,696,419	$0	$310,036
HIV services for people in prisons[3]	$6,263	$33,121	$260,754	$208,616	$13,575
Total	$130,067,484	$13,086,135	$42,345,328	$15,513,102	$31,853,627
**Optimized spending with reduced ART costs**					
ART	$108,435,556	$7,078,622	$20,342,828	$4,967,905	$20,814,492
PMTCT	$799,724	$248,878	$488,518	$35,434	$3,098,392
Treatment support programs	$0	$0	$4,456,810	$0	$0
HIV testing for the general population	$7,476,728	$2,182,089	$4,087,769	$4,076,780	$1,127,551

Country	Colombia	Costa Rica	Dominican Republic	El Salvador	Honduras
Condom distribution/promotion among the general population[1]	$84,661	$1,439,829	$519,396	$386,568	$2,263,555
PrEP[2]	$911,794	$1,083,763	$1,684,858	$1,935,402	$0
HIV self-testing	$821,954	$0	$0	$0	$0
HIV services for MSM[3]	$9,467,163	$574,971	$4,488,498	$1,934,940	$3,234,763
HIV services for transgender women[3]	$212,490	$256,131	$593,830	$86,378	$494,164
HIV services for FSW[3]	$189,919	$188,730	$1,732,749	$1,881,078	$454,526
HIV services for PWID[3]	$430,184	$0	$0	$0	$0
HIV services for migrants or Indigenous populations[3]	$842,792	$0	$3,458,558	$0	$310,036
HIV services for people in prisons[3]	$394,519	$33,121	$491,515	$208,616	$56,148
Total	$130,067,484	$13,086,135	$42,345,328	$15,513,102	$31,853,627

ART, antiretroviral treatment; FSW, female sex workers; MSM, men who have sex with men; N/A, not applicable (not modeled); PMTCT, prevention of mother-to-child transmission; PrEP, pre-exposure prophylaxis; PWID, people who inject drugs.

[1]Condom distribution and social and behavior change communication reaching non-key populations (i.e. not focused on key population groups). Excludes private condom spending in El Salvador ($2,640,581) and Honduras ($8,339,061) which was considered fixed spending and not part of the optimizable budget.

[2]Based on spending on post-exposure prophylaxis (PEP) in El Salvador. Includes both oral and long-acting PrEP in Colombia.

[3]Includes HIV prevention (excluding PrEP) and testing services tailored to key population groups.

Projected epidemic impact of 100% spending optimized with uncertainty bounds

To quantify uncertainty in the model projections, we generated 100 plausible baseline projections by sampling the key calibration parameters: initial HIV prevalence, and force of infection, as well as varying HIV disease progression and mortality rates given in Table A-2. The variation in these parameters was chosen such that the resulting envelope of model projections for estimated new HIV acquisitions, HIV-related deaths and the number of people living with HIV would align to the epidemiological uncertainty in UNAIDS estimates for each country. Each of the scenarios were then applied onto these plausible parameter sets.

Figure C-3. Number of new HIV acquisitions and HIV-related deaths, 2022-2030, under the counterfactual (fixed spending) and 100% spending optimized scenarios by country

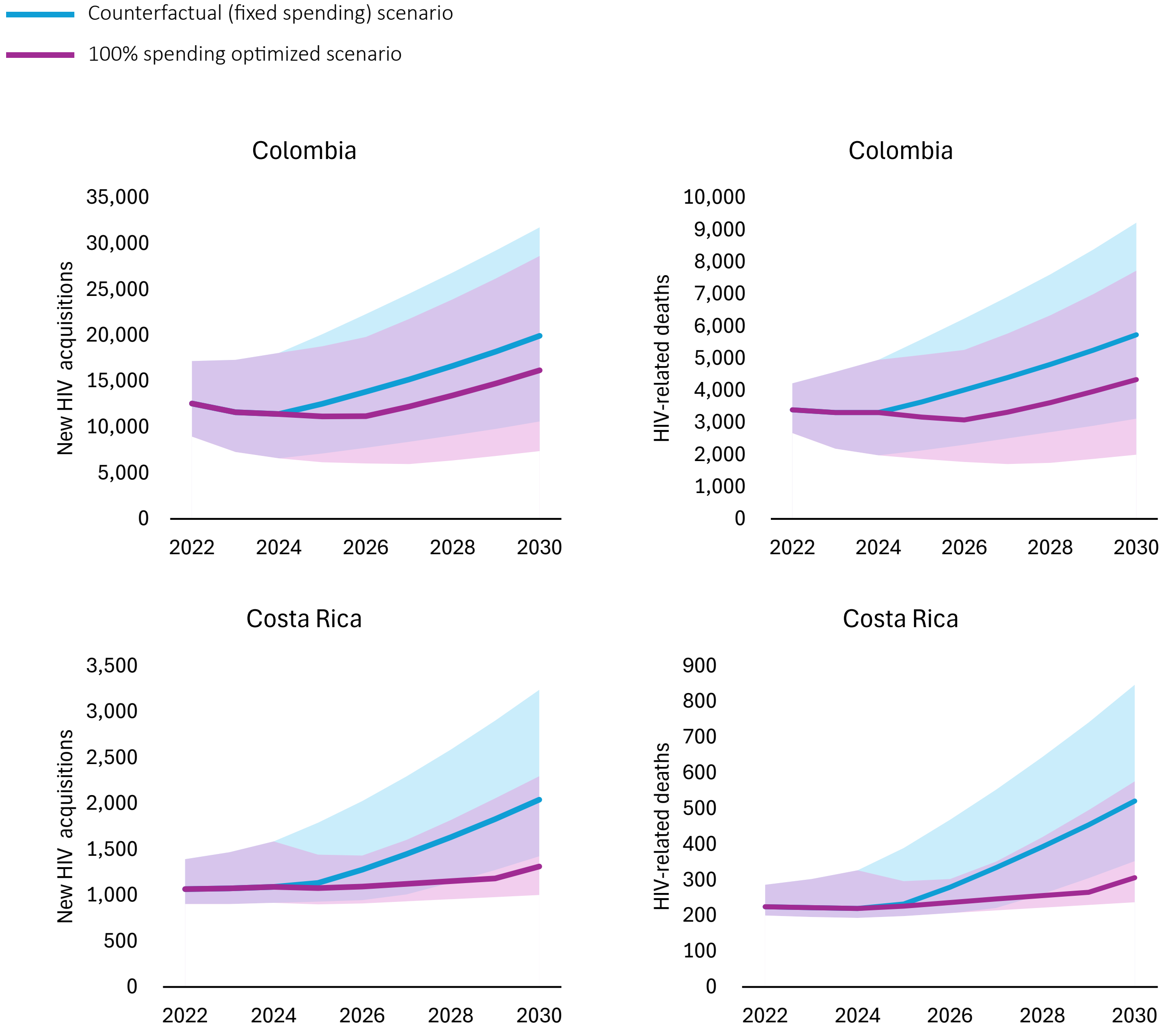

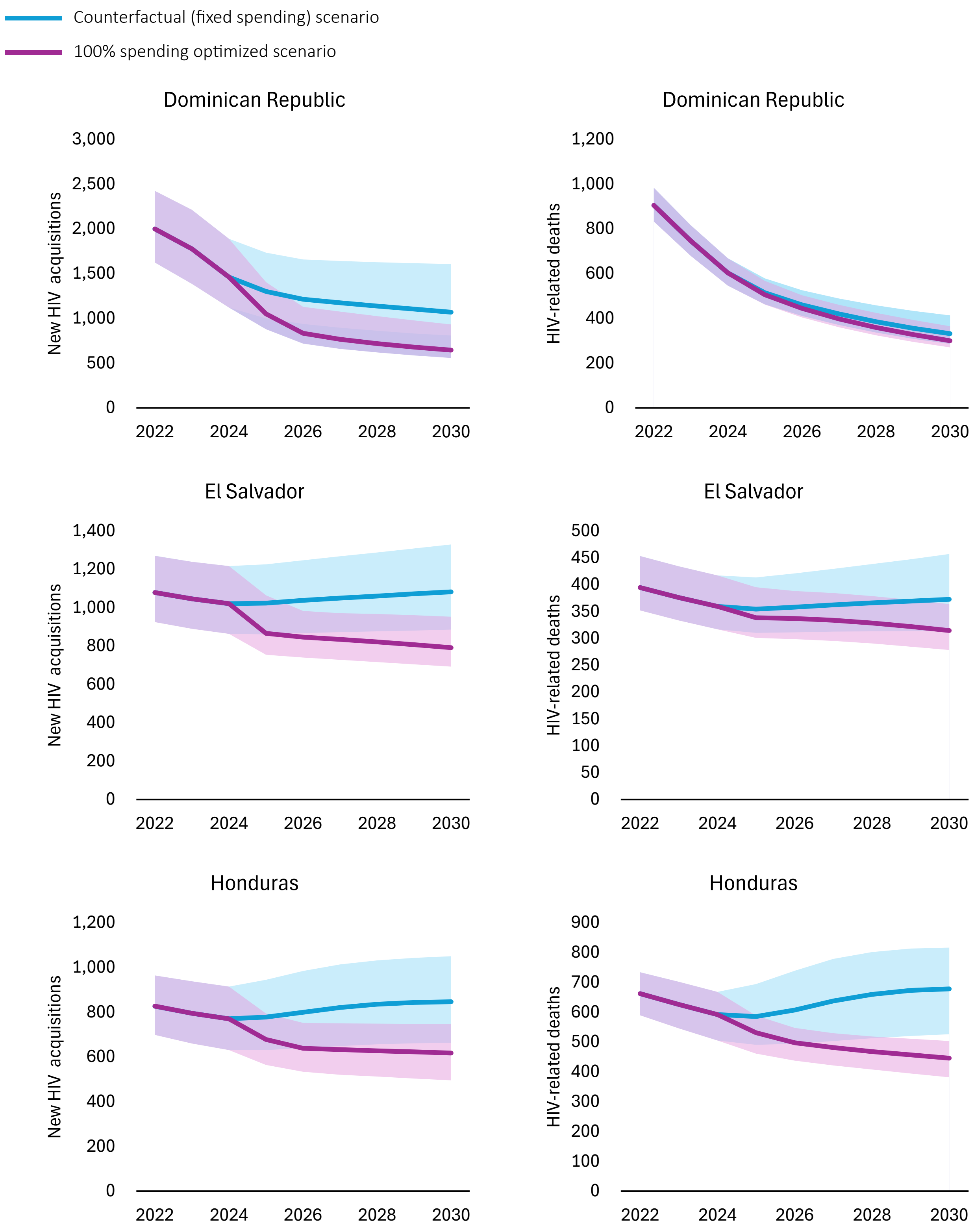
Counterfactual (fixed spending) scenario
100% spending optimized scenario
Dominican Republic
New HIV acquisitions
3,000
2,500
2,000
1,500
1,000
500
0
2022
2024
2026
2028
2030
Dominican Republic
HIV-related deaths
1,200
1,000
800
600
400
200
0
2022
2024
2026
2028
2030
El Salvador
New HIV acquisitions
1,400
1,200
1,000
800
600
400
200
0
2022
2024
2026
2028
2030
El Salvador
HIV-related deaths
500
450
400
350
300
250
200
150
100
50
0
2022
2024
2026
2028
2030
Honduras
New HIV acquisitions
1,200
1,000
800
600
400
200
0
2022
2024
2026
2028
2030
Honduras
HIV-related deaths
900
800
700
600
500
400
300
200
100
0
2022
2024
2026
2028
2030

Projected epidemic impact of 100% spending optimized (reduced ART costs) with uncertainty bounds

Figure C-4. Number of new HIV acquisitions and HIV-related deaths, 2022-2030, under the counterfactual (fixed spending) and 100% spending optimized with reduced ART costs scenarios by country

Counterfactual (fixed spending) scenario

100% spending optimized with reduced ART costs scenario

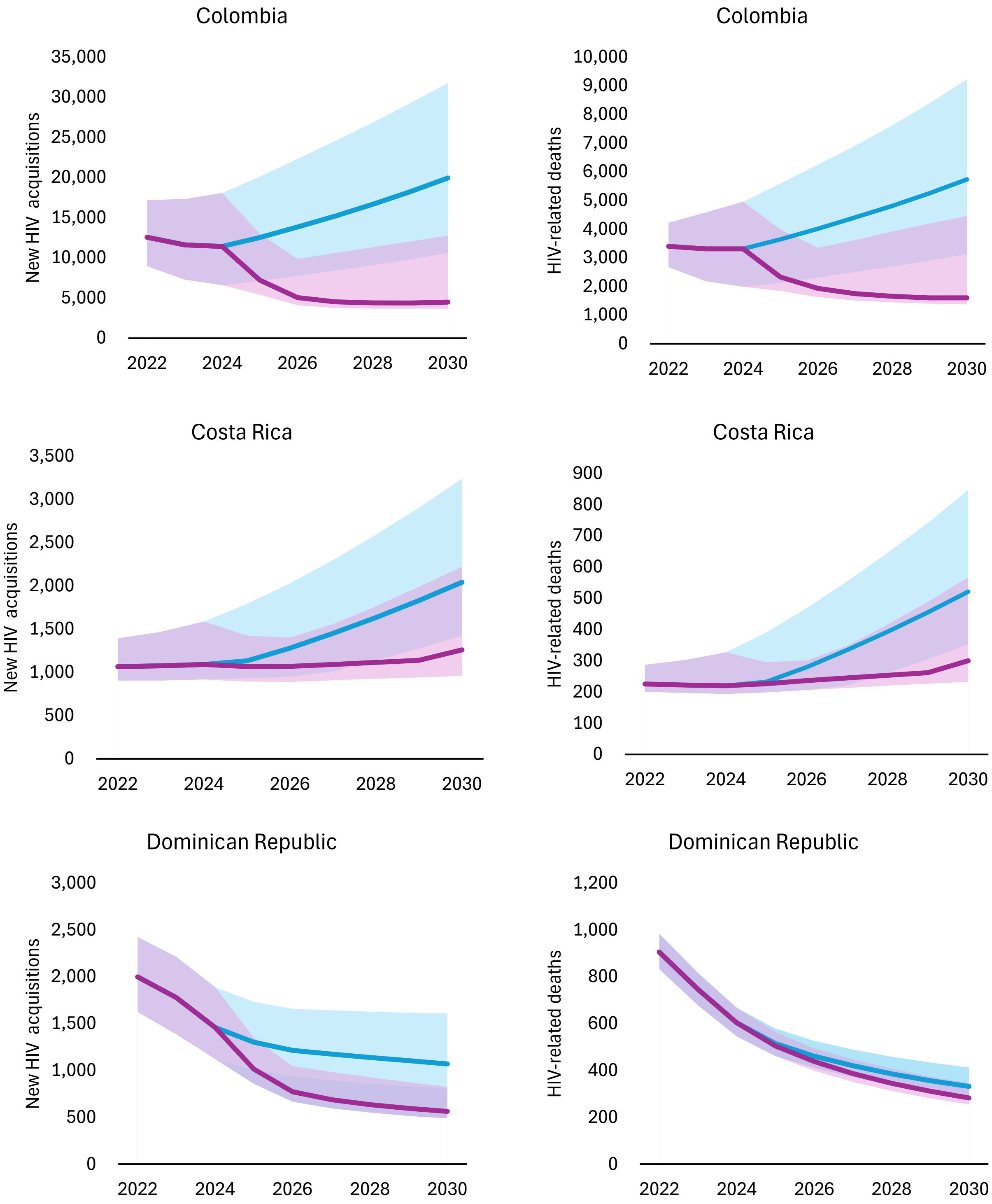

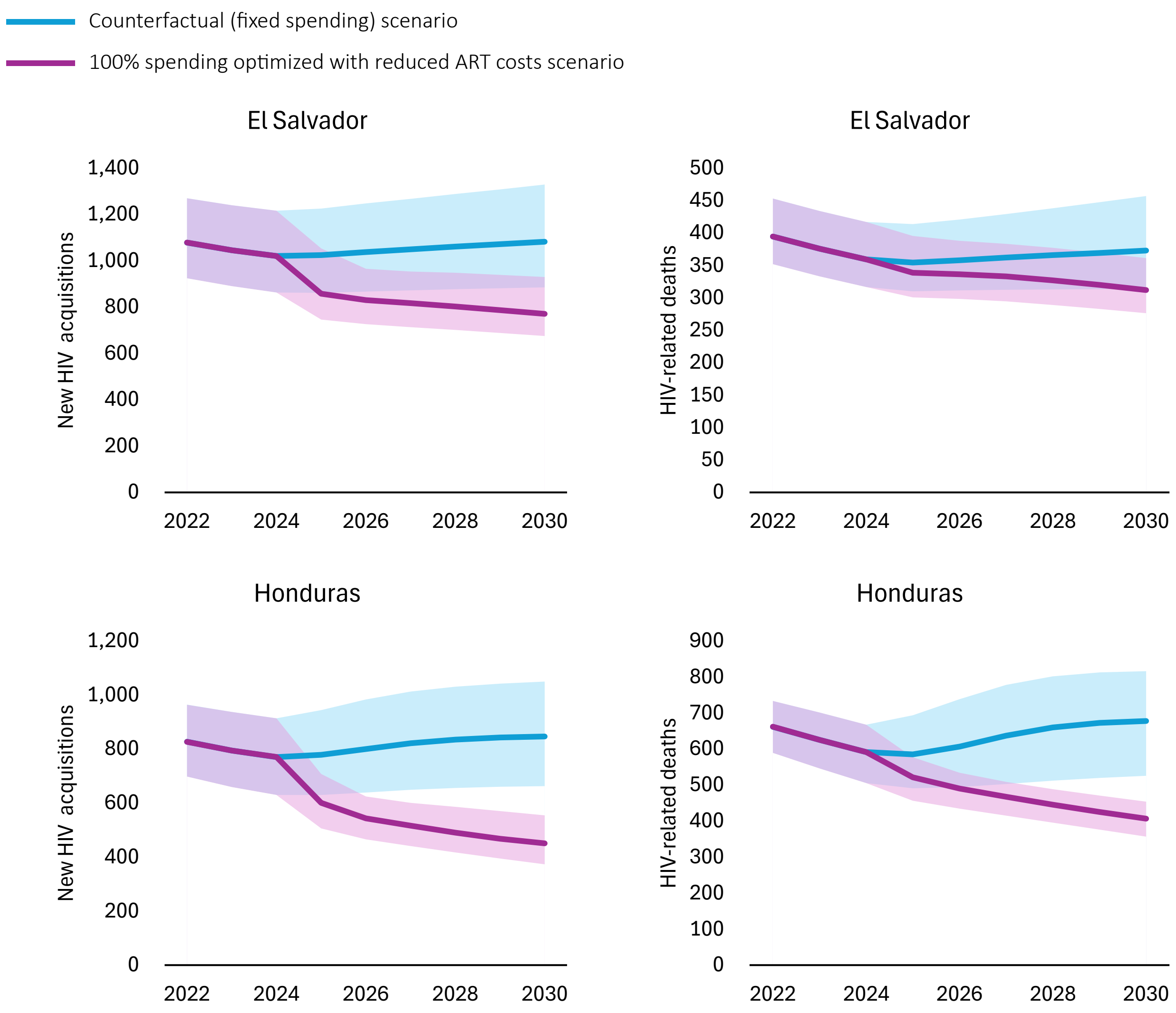

Counterfactual (fixed spending) scenario
100% spending optimized with reduced ART costs scenario
El Salvador
New HIV acquisitions
1,400
1,200
1,000
800
600
400
200
0
2022
2024
2026
2028
2030
El Salvador
HIV-related deaths
500
450
400
350
300
250
200
150
100
50
0
2022
2024
2026
2028
2030
Honduras
New HIV acquisitions
1,200
1,000
800
600
400
200
0
2022
2024
2026
2028
2030
Honduras
HIV-related deaths
900
800
700
600
500
400
300
200
100
0
2022
2024
2026
2028
2030

Sensitivity analysis: Alternative optimization objectives

To perform a budget optimization, an objective needs to be defined that can be maximized or minimized. The Optima Consortium for Decision Science generally recommend using an optimization objective to minimize new HIV acquisitions and HIV-related deaths at a weighting of 1:5, as this produces a balanced optimization, generally aligned to policy objectives [24]. Defining alternative objectives will impact allocation priorities.

Optimizing with an objective to **minimize deaths** alone (Table C-2) prioritizes treatment and treatment support over prevention services, particularly over the short-term, given that it takes longer for prevented infections to lead to prevented deaths.

Table C-2. Change in spending allocation by intervention and country with 100% spending optimized to minimize HIV-related deaths

	Colombia	Costa Rica	Dominican Republic	El Salvador	Honduras
ART	+$10,397,000 (10%)	+$1,778,000 (23%)	+$1,724,000 (7%)	+$1,738,000 (36%)	+$4,933,000 (24%)
PMTCT	No change	No change	No change	No change	No change
Treatment support programs			+$2,089,000 (113%)		
HIV testing for the general population[1]	-$7,477,000 (-50%)	+$27,000 (2%)	-$4,088,000 (-50%)	+$1,765,000 (49%)	-$1,128,000 (-50%)
Condom distribution/promotion among the general population	-$85,000 (-50%)	-$1,440,000 (-50%)	-$519,000 (-50%)	-$387,000 (-50%)	-$2,264,000 (-50%)
PrEP[2]	-$912,000 (-50%)	+$19,000 (6%)	-$114,000 (-50%)	-$1,935,000 (-50%)	
HIV self-testing	-$21 (-50%)				
HIV prevention and testing services for MSM[3]	-$767,000 (-50%)	-$123,000 (-50%)	+$256,000 (13%)	-$627,000 (-50%)	-$683,000 (-50%)
HIV prevention and testing services for transgender women[3]	-$50,000 (-50%)	-$74,000 (-50%)	+$2,000 (1%)	-$86,000 (-50%)	-$81,000 (-50%)
HIV prevention and testing services for FSW[3]	-$190,000 (-50%)	-$154,000 (-50%)	-$771,000 (-50%)	-$259,000 (-50%)	-$455,000 (-50%)
HIV prevention and testing services for PWID	-$68,000 (-50%)				
HIV prevention and testing services for migrants / Indigenous populations	-$843,000 (-50%)		+$1,234,000 (84%)		-$310,000 (-50%)
HIV prevention and testing services for people in prisons	-$6,000 (-50%)	-$33,000 (-50%)	+$187,000 (14594%)	-$209,000 (-50%)	-$14,000 (-50%)

KEY
- Increased spending prioritized
- Spending maintained (±2%)
- Decreased spending
- Not modeled

ART, antiretroviral therapy; FSW, female sex workers; MSM, men who have sex with men; PMTCT, prevention of mother-to-child (vertical) transmission; PrEP, pre-exposure prophylaxis (includes post-exposure prophylaxis in El Salvador); PWID, people who inject drugs. All spending rounded to nearest 1000 and reported in 2024 US$.
[1]General HIV testing modeled to reach all populations in Costa Rica;
[2]Based on post-exposure prophylaxis in El Salvador;
[3]Key-population focused programs exclude testing in Costa Rica.

Optimizing with an objective to **minimize new HIV acquisitions** (Table C-3) prioritizes more prevention services, with a lower allocation for treatment. In this analysis, this is particularly evident in Colombia.

Table C-3. Change in spending allocation by intervention and country with 100% spending optimized to minimize new HIV acquisitions

	Colombia	Costa Rica	Dominican Republic	El Salvador	Honduras
ART	+$4,343,000 (4%)	+$1,656,000 (21%)	+$1,393,000 (5%)	+$1,731,000 (35%)	+$3,292,000 (16%)
PMTCT	No change	+$209,000 (564%)	No change	No change	+$1,580,000 (104%)
Treatment support programs			+$1,245,000 (67%)		
HIV testing for the general population[1]	-$7,477,000 (-50%)	-$680,000 (-50%)	-$4,088,000 (-50%)	-$1,792,000 (-50%)	-$1,128,000 (-50%)
Condom distribution/promotion among the general population	-$85,000 (-50%)	-$1,440,000 (-50%)	-$519,000 (-50%)	-$387,000 (-50%)	-$2,264,000 (-50%)
PrEP[2]	-$912,000 (-50%)	+$513,000 (158%)	+$1,255,000 (550%)	-$1,935,000 (-50%)	
HIV self-testing	-$21 (-50%)				
HIV prevention and testing services for MSM[3]	+$4,873,000 (318%)	-$45,000 (-18%)	+$1,100,000 (57%)	+$1,305,000 (104%)	-$683,000 (-50%)
HIV prevention and testing services for transgender women[3]	+$93,000 (94%)	-$27,000 (-18%)	+$150,000 (66%)	-$86,000 (-50%)	-$20,000 (-12%)
HIV prevention and testing services for FSW[3]	-$190,000 (-50%)	-$154,000 (-50%)	-$243,000 (-16%)	+$1,372,000 (265%)	-$455,000 (-50%)
HIV prevention and testing services for PWID	+$204,000 (149%)				
HIV prevention and testing services for migrants / Indigenous populations	-$843,000 (-50%)		-$597,000 (-41%)		-$310,000 (-50%)
HIV prevention and testing services for people in prisons	-$6,000 (-50%)	-$33,000 (-50%)	+$305,000 (23830%)	-$209,000 (-50%)	-$14,000 (-50%)

KEY
- Increased spending prioritized (blue)
- Spending maintained (±2%) (yellow)
- Decreased spending (red)
- Not modeled (grey)

ART, antiretroviral therapy; FSW, female sex workers; MSM, men who have sex with men; PMTCT, prevention of mother-to-child (vertical) transmission; PrEP, pre-exposure prophylaxis (includes post-exposure prophylaxis in El Salvador); PWID, people who inject drugs. All spending rounded to nearest 1000 and reported in 2024 US$.

[1]General HIV testing modeled to reach all populations in Costa Rica;

[2]Based on post-exposure prophylaxis in El Salvador;

[3]Key-population focused programs exclude testing in Costa Rica.

Optimizing with an objective to **minimize disability-adjusted life years (DALYs)** (Table C-4) provided a similar allocation of spending as optimizing for deaths in these five Latin American and Caribbean countries given that deaths drive HIV-related DALYs. Differences included no prioritization of PrEP, and higher allocation of prevention and testing services for key populations in Dominican Republic.

Table C-4. Change in spending allocation by intervention and country with 100% spending optimized to minimize disability-adjusted life years (DALYs)

	Colombia	Costa Rica	Dominican Republic	El Salvador	Honduras
ART	+$10,397,000 (10%)	+$1,778,000 (23%)	+$1,569,000 (6%)	+$1,565,000 (32%)	+$4,933,000 (24%)
PMTCT	No change	No change	No change	No change	No change
Treatment support programs			+$1,462,000 (79%)		
HIV testing for the general population[1]	-$7,477,000 (-50%)	+$27,000 (2%)	-$4,088,000 (-50%)	+$1,938,000 (54%)	-$1,128,000 (-50%)
Condom distribution/promotion among the general population	-$85,000 (-50%)	-$1,440,000 (-50%)	-$519,000 (-50%)	-$387,000 (-50%)	-$2,264,000 (-50%)
PrEP[2]	-$912,000 (-50%)	+$19,000 (6%)	-$114,000 (-50%)	-$1,935,000 (-50%)	
HIV self-testing	-$21 (-50%)				
HIV prevention and testing services for MSM[3]	-$767,000 (-50%)	-$123,000 (-50%)	+$670,000 (35%)	-$627,000 (-50%)	-$683,000 (-50%)
HIV prevention and testing services for transgender women[3]	-$50,000 (-50%)	-$74,000 (-50%)	+$158,000 (70%)	-$86,000 (-50%)	-$81,000 (-50%)
HIV prevention and testing services for FSW[3]	-$190,000 (-50%)	-$154,000 (-50%)	-$771,000 (-50%)	-$259,000 (-50%)	-$455,000 (-50%)
HIV prevention and testing services for PWID	-$68,000 (-50%)				
HIV prevention and testing services for migrants / Indigenous populations	-$843,000 (-50%)		+$1,337,000 (91%)		-$310,000 (-50%)
HIV prevention and testing services for people in prisons	-$6,000 (-50%)	-$33,000 (-50%)	+$296,000 (23153%)	-$209,000 (-50%)	-$14,000 (-50%)

KEY
- Increased spending prioritized
- Spending maintained (±2%)
- Decreased spending
- Not modeled

ART, antiretroviral therapy; FSW, female sex workers; MSM, men who have sex with men; PMTCT, prevention of mother-to-child (vertical) transmission; PrEP, pre-exposure prophylaxis (includes post-exposure prophylaxis in El Salvador); PWID, people who inject drugs. All spending rounded to nearest 1000 and reported in 2024 US$.

[1]General HIV testing modeled to reach all populations in Costa Rica;

[2]Based on post-exposure prophylaxis in El Salvador;

[3]Key-population focused programs exclude testing in Costa Rica.

References

1. Kerr CC, Stuart RM, Gray RT, Shattock AJ, Fraser-Hurt N, Benedikt C, et al. Optima: A Model for HIV Epidemic Analysis, Program Prioritization, and Resource Optimization. J Acquir Immune Defic Syndr. 2015;69(3):365-76.
2. United Nations. World Population Prospects: 2024 revision: United Nations Department of Economic and Social Affairs, Population Division; 2024. Available from: https://population.un.org/wpp/.
3. Fernández B, Yajaira D. Comportamiento sexual y prevalencia de VIH en hombres que tienen relaciones sexuales con hombres en tres ciudades de Colombia: Bogotá, Medellín y Santiago de Cali. Medellín: Universidad CES y Empresa Nacional Promotora del Desarrollo Territorial (ENTerritorio); 2019.
4. Estudio de prevalencia de VIH, Hepatitis B, Hepatitis C, Sífilis, y comportamientos asociados en las personas que se inyectan drogas en las ciudades de Armenia y Cúcuta en el 2021: Universidad CES y Ministerio de Justicia y del Derecho; 2021.
5. Estudio de prevalencia de VIH, Hepatitis B, Hepatitis C, Sífilis, y comportamientos asociados en las personas que se inyectan drogas: Bogotá, Medellín, Santiago de Cali y el Área Conurbada de Pereira y Dosquebradas, 2021: Fundación Salutia- Centro de estudios, innovación e investigación en salud; 2022.
6. Censo de Habitantes de la Calle (CHC) 2021: Comunicado de prensa. Bogotá, D.C.: Departamento Administrativo Nacional de Estadística (DANE); 2021.
7. Fernández B, Yajaira D. Vulnerabilidad al VIH y prevalencia de VIH en mujeres transgénero en tres ciudades de Colombia : Bogotá, Medellín y Santiago de Cali. Medellín: Universidad CES y Empresa Nacional Promotora del Desarrollo Territorial (ENTerritorio); 2019.
8. Informe de migrantes venezolanas(os) en Colombia corte 31 de diciembre de 2024: Migración Colombia; 2025 [cited 2025 Sept 8]. Available from: https://www.migracioncolombia.gov.co/infografias-migracion-colombia/informe-de-migrantes-venezolanasos-en-colombia.
9. Red Somos, Ministry of Health and Social Protection, Johns Hopkins University. Biobehavioral survey of HIV, syphilis, and health status among Venezuelans living in Colombia: Final report. Bogotá and Baltimore: Johns Hopkins University; 2022.
10. DANE. Proyecciones de población y estudios demográficos. Updated July 18, 2025. Bogotá D.C., Colombia: Departamento Administrativo Nacional de Estadística (DANE); 2025 [cited 2025 13 Oct].
11. Encuesta de comportamiento sexual y prevalencia de VIH e ITS y estimación del tamaño de poblaciones clave: mujeres trans, hombres que tienen relaciones sexuales con hombres y mujeres trabajadoras sexuales de la Gran Area Metropolitana. San José, Costa Rica: Ministerio de Salud; 2018.
12. Actualización sobre las Definiciones y Dimensionamiento de las Poblaciones Clave. Santo Domingo, República Dominicana: Asesoría y Servicios en Salud Internacional; 2019.
13. CONAVIHSIDA. Cuarta Encuesta de Vigilancia de Comportamiento con Vinculación Serológica: Resultados Prevalencia y Uso de Condón. Santo Domingo: Consejo Nacional para el VIH y SIDA (CONAVIHSIDA); 2021.
14. Centro de Estudios Sociales y Demográficos (CESDEM), ICF International. Encuesta Demográfica y de Salud (ENDESA) 2013. Santo Domingo, República Dominicana: CESDEM y ICF International; 2014.
15. CONAVIHSIDA. Plan Estratégico Nacional (PEN 2021-2024). Santo Domingo: Consejo Nacional para el VIH y el SIDA (CONAVIHSIDA); 2021.
16. CONAVIHSIDA. Segunda Encuesta de Vigilancia de Comportamiento con Vinculación Serológica en Poblaciones Claves: Gais, Trans y Hombres que tienen Sexo con Hombres (GTH), Trabajadoras Sexuales (TRSX), Usuarios de Drogas (UD). República Dominicana, Año 2012. Santo Domingo: Consejo Nacional para el VIH y SIDA (CONAVIHSIDA); 2014.

17. Oficina Nacional de Estadística (ONE). Segunda Encuesta Nacional de Inmigrantes, ENI-2017. Santo Domingo: Oficina Nacional de Estadística; 2018.
18. CONAVIHSIDA. Tercera Encuesta de Vigilancia de Comportamiento con Vinculación Serológica en Poblaciones Claves: Gays, y Hombres que tienen sexo con hombres (GH), mujeres transgénero (TRANS), trabajadoras sexuales (TRSX), migrantes de nacionalidad Haitiana (MH) y personas que viven con VIH (PVVIH). Santo Domingo: Consejo Nacional para el VIH y SIDA (CONAVIHSIDA); 2018.
19. Ministerio de Salud de Honduras. Encuesta Centroamericana de Vigilancia del Comportamiento enfocada a trabajadoras sexuales, hombres que tienen sexo con otros hombres, población trans y población Garífuna, 2012. Tegucigalpa: Ministerio de Salud de Honduras; 2012.
20. Pérez Á, Soto J, Garavito L, Álvarez C, Barlizagela M. Un paquete de atención integral para personas en situación de migración irregular o pendular con VIH. Proyecto de Sostenibilidad del Sistema de Salud Local (LHSS) bajo el IDIQ de los Sistemas Integrados de Salud de USAID. Rockville, MD: Abt Associates; 2022.
21. ALZAK Foundation. Actualización de evaluación del impacto presupuestal de la estrategia PrEP y del autotest para la prevención de la infección por VIH en Colombia 2020. Cartagena, Colombia: ALZAK Foundation; 2020.
22. AVAC. PrEP Price Comparison2025 [cited 2025 Oct 24]. Available from: https://avac.org/resource/infographic/prep-price-comparison/.
23. Informe de gestión 2021-2022 Colombia: AID for AIDS Colombia; 2023.
24. Optima HIV User Guide Volume II: Software Reference Manual: Optima Consortium for Decision Science; 2024. Available from: https://optimamodel.com/docs/Optima%20HIV%20User%20Guide%20Reference%20Manual.pdf.
